# Evaluation of Anti-HIV-1 HSPC-Based Gene Therapy with Negative Selection Marker in Pigtail Macaques

**DOI:** 10.3390/v18101133

**Published:** 2026-10-13

**Authors:** Jian Zhang, Jason Murray, Wenli Mu, Surya Venugopal, Ella Yim, Teresa Einhaus, Stefan Radtke, Christopher W. Peterson, Hans-Peter Kiem, Dong Sung An

**Affiliations:** 1 University of California Los Angeles (UCLA) AIDS Institute, Los Angeles, CA 90024, USA; jianyinzhang@sonnet.ucla.edu (J.Z.); wmu@mednet.ucla.edu (W.M.); suryavenugopal@sonnet.ucla.edu (S.V.); 2 UCLA Joe C. Wen School of Nursing, UCLA, Los Angeles, CA 90095, USA; 3 Stem Cell and Gene Therapy Program, Fred Hutchinson Cancer Center, Seattle, WA 98109, USA; jmurray2@fredhutch.org (J.M.); teinhaus@fredhutch.org (T.E.); sradtke@fredhutch.org (S.R.); cwpeters@fredhutch.org (C.W.P.); hkiem@fredhutch.org (H.-P.K.); 4 Department of Medicine, Division of Hematology and Oncology, David Geffen School of Medicine, UCLA, Los Angeles, CA 90095, USA; 5 Department of Microbiology, Immunology, and Molecular Genetics, UCLA, Los Angeles, CA 90095, USA; ellayim@g.ucla.edu; 6 Department of Medicine, University of Washington, Seattle, WA 98195, USA

**Keywords:** HIV-1, SHIV, hematopoietic stem and progenitor cell (HSPC) gene thCherapy, lentiviral vector, chimeric antigen receptor (CAR), CCR5 shRNA, C46 fusion inhibitor, huEGFRt, Cetuximab, non-human primate (NHP)

## Abstract

Despite significant medical progress, HIV-1 remains a critical global health crisis. While combination antiretroviral therapy (cART) effectively suppresses viremia, it is not curative due to the persistence of latent viral reservoirs. Autologous hematopoietic stem and progenitor cell (HSPC) gene therapy offers a promising avenue toward sustained, drug-free remission. In this study, we investigated the feasibility and safety of a multifunctional “defend and attack” HSPC gene therapy strategy in the pigtail macaque (*Macaca nemestrina*) model. The species-optimized lentiviral vector, PM-MNDU-anti-HIV-1-huEGFRt, carries four therapeutic payloads: a pigtail macaque-specific CCR5 shRNA (PM-CCR5sh1005), a CD4-based chimeric antigen receptor (D1D2CAR), a C46 fusion inhibitor peptide, and a truncated human epidermal growth factor receptor (huEGFRt) serving as a cell-surface tracking marker and anti-EGFR antibody Cetuximab (CTX)-mediated negative selection marker. Following busulfan conditioning, autologous transplantation of vector-modified HSPCs in two pigtail macaques resulted in safe hematopoietic recovery. Both animals achieved sustained, multilineage gene marking in peripheral blood and lymphoid tissues without adverse effects. In one animal, upon Simian–Human Immunodeficiency Virus (SHIV) challenge, vector-modified cells underwent robust, antigen-driven multilineage expansion across both lymphoid and myeloid compartments, which contracted during cART-induced viral suppression and rapidly rebounded following treatment interruption. Administration of CTX demonstrated partial, transient reduction in huEGFRt^+^ cells, accompanied with a reciprocal increase in plasma viral load. These findings support the feasibility of evaluating a multi-pronged anti-HIV HSPC gene therapy strategy with a negative selection marker in a non-human primate (NHP) model.

## 1. Introduction

Despite significant medical progress, HIV-1 remains a critical global health crisis, with global prevalence approaching 41 million cases [1]. While combination antiretroviral therapy (cART) effectively suppresses viremia and extends life expectancy, it is not curative [2,3,4,5]. The persistence of latent viral reservoirs necessitates lifelong adherence, presenting ongoing challenges regarding cumulative drug toxicity, financial cost, administrative barriers, and chronic inflammatory complications [6,7,8,9,10]. To date, sustained drug-free remission has been achieved in seven patients via allogeneic hematopoietic stem cell transplantation using donor graft sources harboring the naturally occurring CCR5 Δ32 mutation [11,12,13,14,15,16,17,18]. In one patient, sustained drug-free remission was achieved after wild-type CCR5 stem cell transplantation [19]. The extreme scarcity of compatible CCR5 Δ32 mutation donors renders this approach unscalable for the vast majority of people living with HIV.

To overcome these limitations, autologous hematopoietic stem and progenitor cell (HSPC) gene therapy has emerged as a transformative alternative by genetically engineering a patient’s own stem cells to express anti-HIV payloads. To address the dual challenges of viral persistence and host cell vulnerability, we previously engineered a multifunctional “defend and attack” lentiviral vector [20]. This platform utilizes a triple-gene payload designed to intercept the HIV-1 life cycle at multiple stages: a non-toxic short hairpin RNA (shRNA 1005) to downregulate the CCR5 co-receptor to protect cells from R5 tropic HIV-1 entry [21,22,23], and a membrane-anchored C46 peptide that inhibits HIV-1 gp41-mediated fusion [23,24,25,26,27]. CCR5sh1005 and C46 can synergistically protect cells from HIV-1 entry and inhibit both R5-tropic and X4-tropic HIV-1 [25]. Beyond these defensive modalities, the vector incorporates a CD4-based chimeric antigen receptor (CAR)—specifically the D1D2CAR 4-1BB variant—to actively eliminate infected cells by targeting the viral gp120 envelope protein [28]. Together, CCR5sh1005, C46, and D1D2CAR can inhibit three different steps in HIV-1 infection, thereby protecting HIV-1-susceptible cells while targeting HIV-1 infected cells.

The clinical application of HSPC gene therapy also demands robust safety mechanisms to mitigate potential vector- or CAR-mediated toxicities. While lentiviral vectors exhibit a favorable safety profile compared to gammaretroviruses, they maintain a low but non-negligible risk of insertional oncogenesis [29,30,31,32,33,34]. Furthermore, potent CAR-T cell responses carry inherent risks of hyper-inflammatory complications, such as cytokine release syndrome or off-target reactivity [35,36,37,38]. Incorporating a controllable safety feature into the vector architecture provides an essential fail-safe mechanism. To this end, our vector includes a truncated, non-functional human epidermal growth factor receptor (huEGFRt) [39,40]. Serving a dual purpose, huEGFRt acts as a cell-surface selection marker and as a pharmacologically inducible safety switch, permitting the targeted antibody-dependent cellular cytotoxicity (ADCC)-mediated elimination of transduced cells using the FDA-approved monoclonal antibody Cetuximab.

While our previous studies in humanized bone marrow/liver/thymus (BLT) mice provided proof-of-concept for this approach, small animal models are inherently limited in their ability to recapitulate the scale and complexity of human hematopoiesis, long-term stem cell self-renewal, and tissue-specific immune reconstitution [41,42,43]. Clinical translation of HSPC-based therapies therefore requires evaluation in a physiologically relevant large animal model. Non-human primates (NHPs), particularly pigtail macaques (*Macaca nemestrina*), provide an essential translational platform, established utility in autologous HSPC transplantation, and susceptibility to Simian–Human Immunodeficiency Virus (SHIV) infection [27,44,45].

In the present study, we extended our “defend and attack” HSPC gene therapy strategy into the pigtail macaque model using a species-optimized lentiviral vector to investigate the feasibility, safety, and dynamics of a multi-pronged HSPC gene therapy. We evaluated the efficiency of this vector in gene-modifying CD34^+^ HSPCs—including long-term repopulating fractions—and assessed the long-term engraftment, tissue distribution, and in vivo dynamics of their progeny in the context of SHIV infection, cART intervention, and cART interruption. Furthermore, we evaluated the utility of a truncated human epidermal growth factor receptor (huEGFRt) as both a cell-surface tracking tag and a pharmacologically inducible negative selection marker via Cetuximab (CTX)-mediated depletion in vivo.

## 2. Materials and Methods

### 2.1. Ethics Statement

This study was carried out in strict accordance with the recommendations in the Guide for the Care and Use of Laboratory Animals of the National Institutes of Health, and was approved by the Institutional Animal Care and Use Committees (IACUC) of the Fred Hutchinson Cancer Center and the University of Washington (Protocol #3235-04). All pigtail macaques (*Macaca nemestrina*) were housed at the Washington National Biomedical Research Center (WaNBRC) under conditions approved by the Association for Assessment and Accreditation of Laboratory Animal Care (AAALAC) International. Standard monitoring procedures included twice-daily observation by animal technicians for basic husbandry parameters, in addition to daily examinations by veterinary staff. Housing conditions complied with Animal Welfare Act regulations and NIH standards. Environmental enrichment included social grouping when appropriate, perches, toys, food treats, and foraging activities.

Animals were fed twice daily and fasted for 14 h before sedation or anesthetic procedures. Sedation was performed using ketamine hydrochloride and/or Telazol, with adjunctive anesthetic or sedative agents administered as clinically indicated. Physiologic monitoring during procedures included tracking heart rate, respiratory rate, blood oxygen saturation (SpO_2_), blood pressure, and body temperature. Adequate depth of anesthesia was verified by loss of palpebral and toe-pinch reflexes. Post-procedural analgesia (buprenorphine with meloxicam or sustained-release buprenorphine) was administered as prescribed under veterinary guidance for a minimum of 48 h after the procedures and could be extended at the discretion of the clinical veterinarian based on clinical signs. Euthanasia was performed in accordance with AVMA guidelines by inducing deep surgical anesthesia with ketamine followed by an overdose of intravenous sodium pentobarbital.

### 2.2. CD34^+^ HSPC Mobilization and Enrichment

Autologous CD34^+^ HSPCs were mobilized into peripheral blood via recombinant human granulocyte colony-stimulating factor (G-CSF; 50 μg/kg/day subcutaneous (SC) for 5 days) and plerixafor (AMD3100; 1 mg/kg SC on the night of Day 4). Apheresis was conducted on Day 5 using a Spectra Optia apheresis system (Terumo BCT, Lakewood, CO, USA). Harvested cell suspensions underwent ammonium chloride red blood cell lysis. Leukocytes were incubated for 25 min with a custom-produced 12.8 IgM anti-CD34 monoclonal antibody (Fred Hutch, Seattle, WA, USA), washed, and incubated for an additional 25 min with anti-IgM magnetic microbeads (Miltenyi Biotec, Bergisch Gladbach, Germany). The cell suspension was passed through magnetic LS Columns (Miltenyi Biotec) according to manufacturer instructions. Enriched CD34^+^ HSPCs were primed overnight in StemSpan medium (STEMCELL Technologies, Vancouver, BC, Canada) supplemented with penicillin–streptomycin (100 U/mL) (Gibco, Waltham, MA, USA), and 100 ng/mL each of recombinant human stem cell factor (SCF; PeproTech, Rocky Hill, NJ, USA), thrombopoietin (TPO; PeproTech), and FMS-like tyrosine kinase 3 ligand (FLT3-L; Miltenyi Biotec).

### 2.3. Lentiviral Vector Manufacturing and Transduction

VSV-G pseudotyped, research-grade self-inactivating lentiviral vectors were produced by the Fred Hutch Vector Production Core (Director: H.-P. Kiem) via transient transfection of HEK293T cells. Vector titers were determined by transduction of HEK293T cells followed by flow cytometric evaluation of huEGFRt or qPCR-based genomic vector copy number (VCN) quantification. Primed CD34^+^ HSPCs were transduced twice over a 48 h culture period on Retronectin-coated plates (20 μg/mL; Takara Bio, Kusatsu, Japan) in the presence of 4 μg/mL protamine sulfate.

### 2.4. Autologous Transplantation and Busulfan Conditioning

**Figure 1:**
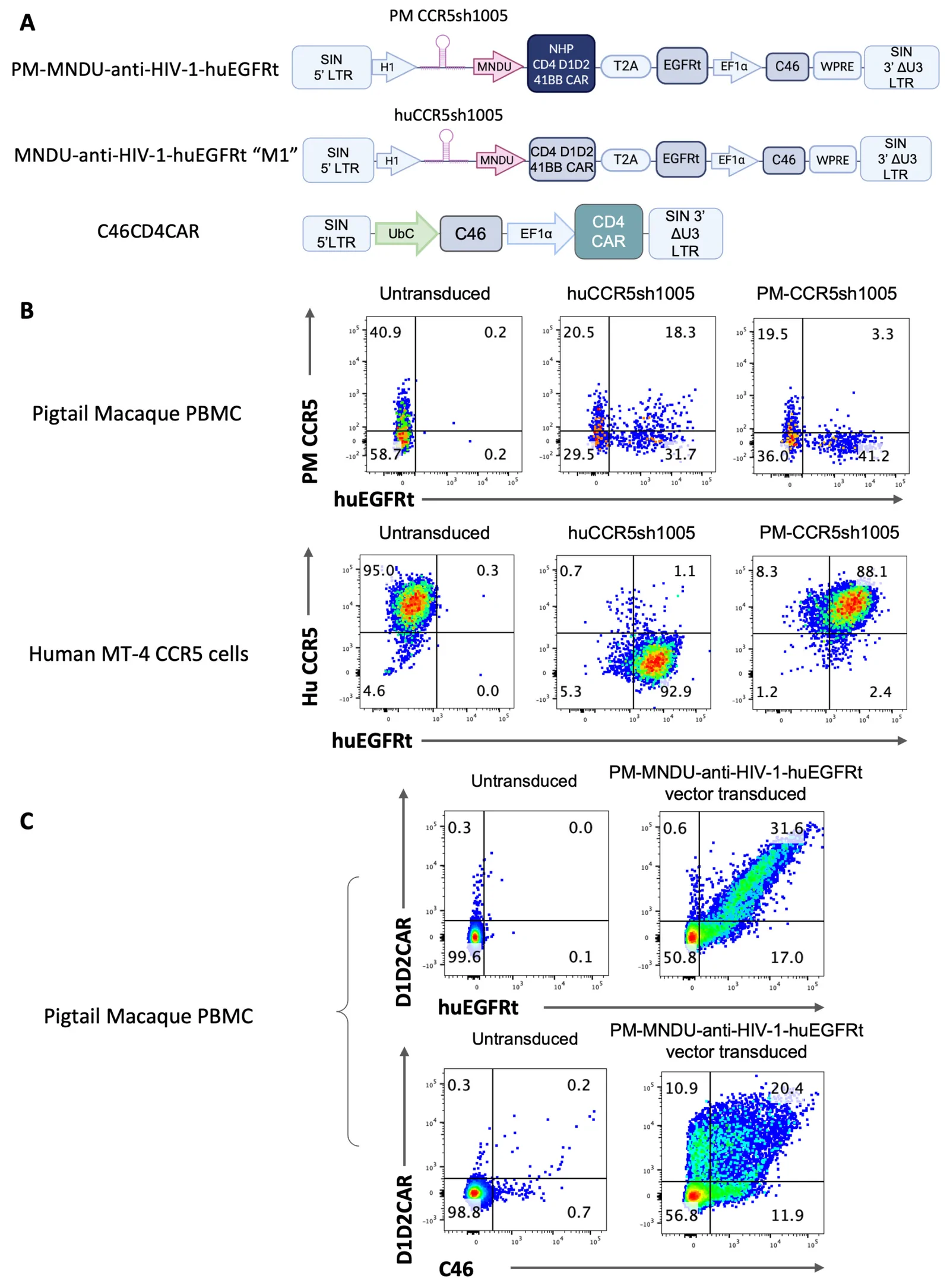
Characterization and species-specific validation of the PM-MNDU-anti-HIV-1-huEGFRt multicistronic lentiviral vector. (**A**) Schematic representation of lentiviral vector constructs. Schematic diagrams illustrating the structural organization of the lentiviral vectors. PM-MNDU-anti-HIV-1-huEGFRt is a non-human primate (NHP)-adapted vector expressing an shRNA targeting pigtail macaque CCR5 (PMCCR5sh1005), an NHP-adapted CD4-based chimeric antigen receptor with a 4-1BB co-stimulatory domain (D1D2CAR 4-1BB), a membrane-anchored C46 fusion inhibitor, and a truncated human epidermal growth factor receptor (huEGFRt) serving as a tracking marker and safety switch. MNDU-anti-HIV-1-huEGFRt (“M1”) is a human-adapted vector expressing an shRNA targeting human CCR5 (huCCR5sh1005), original D1D2CAR 4-1BB, C46, and huEGFRt. The C46CD4CAR control vector expresses C46 alongside a full-length CD4-based CAR. Abbreviations: SIN 3′ ΔU3 LTR, self-inactivating 3′ long terminal repeat with deleted U3 promoter/enhancer regions; H1, H1 RNA polymerase III promoter; MNDU, engineered murine leukemia virus (MuLV) long terminal repeat promoter; T2A, 2A self-cleaving peptide; EF1 alpha, human elongation factor 1-alpha promoter; WPRE, woodchuck hepatitis virus posttranscriptional regulatory element; UbC, human ubiquitin C RNA polymerase II promoter. (**B**) Species-specific downregulation of CCR5 cell-surface expression. PM-CCR5sh1005 mediated efficient CCR5 knockdown in pigtail macaque PBMCs at day 7 post-transduction compared to huCCR5sh1005. Conversely, huCCR5sh1005 downregulated CCR5 in the human MT-4 CCR5 cell line at day 12 post-transduction, whereas PM-CCR5sh1005 did not. Cell-surface expression of CCR5 and huEGFRt was evaluated by fluorophore-conjugated antibody staining and flow cytometry. (**C**) Multicistronic co-expression of D1D2CAR, huEGFRt, and C46 transgenes in transduced pigtail macaque PBMCs, quantified via flow cytometry.

Prior to infusion, subjects received a reduced-intensity myeloablative busulfan conditioning regimen consisting of four daily intravenous doses of 3.5 mg/kg/day (total cumulative dose: 14 mg/kg) spanning Days −4 to −1. Busulfan plasma pharmacokinetics were monitored to confirm target systemic clearance profiles. On Day 0, transduced HSPC infusion products were thawed at 37 °C and re-infused intravenously. Subject Z16052 received 4.87 × 10^7^ PM-MNDU-anti-HIV-1-huEGFRt-transduced autologous CD34^+^ cells (1.74 × 10^5^ CD34^+^CD90^+^CD45RA^−^ cells/kg). Subject Z21191 received a co-infusion mixture comprising 1.34 × 10^7^ total PM-MNDU-anti-HIV-1-huEGFRt-transduced CD34^+^ cells (8.84 × 10^5^ CD34^+^CD90^+^CD45RA^−^ cells/kg) and 1.62 × 10^7^ total C46CD4CAR-transduced CD34^+^ cells (9.86 × 10^5^ CD34^+^CD90^+^CD45RA^−^ cells/kg). C46CD4CAR is a vector that expresses C46 by the ubiquitin C promoter and full-length CD4CAR by eukaryotic translation elongation factor 1 alpha promoter (Figure 1A) [27]. To assess integrated vector copy number (VCN) in the infusion products without interference from non-integrated plasmid or episomal vector DNA, an aliquot of transduced CD34^+^ HSPCs was maintained in ex vivo culture for 6 days prior to genomic DNA isolation and quantitative PCR analysis.

### 2.5. PBMC Isolation

Peripheral blood mononuclear cells (PBMC) were isolated from pigtail macaque blood by Ficoll-Paque density gradient centrifugation (Cytiva, Marlborough, MA, USA). Isolation from pigtail macaque blood consistently yielded a minor population of co-purified residual granulocytes (~1% of PBMC) that was identified by flow cytometry.

### 2.6. Flow Cytometry

Single-cell suspensions from peripheral blood, bone marrow aspirates, and homogenized lymph node tissues were stained with fluorophore-conjugated antibodies. Fluorophore panels included the following: CD34-APC (clone 563; BD Biosciences, Franklin Lakes, NJ, USA), CD45-V450 (clone D058-1283; BD Biosciences), CD90-PE-Cy7 (clone 5E10; BD Biosciences), CD45RA-APC-H7 (clone 5H9; BD Biosciences), CD3-AF700 (clone SP34-2; BD Biosciences), CD20-APC (clone 2H7; BD Biosciences), CD11b-PerCP-Cy5.5 (clone ICRF44; BioLegend, San Diego, CA, USA), CD14-ECD (clone RM052; Beckman Coulter, Indianapolis, IN, USA), CD14-PE-Cy7 (clone M5E2, BD Biosciences), CD4-AF700 (clone L200, BD Biosciences), CD3-BV786 (Clone SP34-2, BD Biosciences), CD8-BV605 (clone RPA-T8, BioLegend), and NKG2a-APC (clone Z199; Beckman Coulter). D1D2CAR and huEGFRt expression was assessed using CD4-FITC (clone RPA-T4; Invitrogen, Waltham, MA, USA) and EGFR-PE (Cetuximab Biosimilar; R&D Systems, Minneapolis, MN, USA). C46 expression was assessed using anti-HIV-1 gp41 mAb (2F5; BEI Resources, Manassas, VA, USA), followed by secondary staining with an anti-human IgG-PE secondary antibody (Jackson ImmunoResearch, West Grove, PA, USA). Stained cells were fixed in 1–2% paraformaldehyde and acquired on LSRFortessa or FACSymphony A5 flow cytometers (BD Biosciences). Data were analyzed using FlowJo software v10.8 (Tree Star, Ashland, OR, USA).

### 2.7. Quantification of Vector DNA Marking by Digital PCR (dPCR)

Genomic DNA was extracted from cell pellets using the QIAamp DNA Blood Mini Kit or DNeasy Blood & Tissue Kit (Qiagen, Hilden, Germany). Absolute vector copy numbers (VCN) per cell were quantified using a QuantStudio Absolute Q Digital PCR System (Thermo Fisher Scientific, Waltham, MA, USA). Thermocycling parameters were as follows: 96 °C for 10 min, followed by 42 cycles of 98 °C for 15 s, 60 °C for 2 min, and a last extension of 60 °C for 2 min. PM-MNDU-anti-HIV-1-huEGFRt was amplified using custom primers/probes targeting the MNDU promoter (Assay ID: APT2JED; Thermo Fisher). C46CD4CAR was detected using primers/probes targeting the unique CD4-CD3ζ junction (Assay ID: APU7DYA). VCN values were normalized to the pigtail macaque reference gene RPP30 (Assay ID: APPRVY9).

### 2.8. SHIV Infection, Combination Antiretroviral Therapy (cART), and Viral Load Analysis

Subjects Z16052 and Z21191 were challenged intravenously with 16700 TCID_50_ of SHIV-1157-ipd3N4 at 441 and 266 days post-transplantation, respectively. Combination antiretroviral therapy (cART) consisted of tenofovir disoproxil fumarate (5.1 mg/kg), emtricitabine (40 mg/kg) (kindly provided by Gilead Sciences, Foster City, CA, USA), and dolutegravir (2.5 mg/kg) (donated by Viiv Healthcare, Research Triangle, NC, USA). Plasma SHIV RNA copies were quantified via quantitative reverse transcription PCR (qRT-PCR) with a lower limit of detection (LLOD) of 20 RNA copies/mL.

### 2.9. Cetuximab-Mediated In Vivo Negative Selection

Following cART withdrawal in subject Z21191, pharmaceutical-grade Cetuximab (Erbitux; Eli Lilly, Indianapolis, IN, USA) was administered intravenously. The dosing schedule comprised three initial weekly infusions of 4 mg/kg followed by a fourth dose of 5 mg/kg. Clinical monitoring for safety and adverse reactions was conducted 2–3 times daily.

### 2.10. Tissue Collection and Processing

At study endpoints or necropsy, tissue specimens (spleen, mesenteric, axillary, iliac, submandibular, and inguinal lymph nodes, and thymus) were harvested immediately without pre-perfusion and stored in MACS Tissue Storage Solution (Miltenyi Biotec). Tissues were mechanically disaggregated into single-cell suspensions for flow cytometric analysis and dPCR VCN quantification. Bone marrow CD34^+^CD90^+^ HSPCs were immunomagnetically enriched from total bone marrow mononuclear cells as described above.

## 3. Results

### 3.1. Characterization of a Pigtail Macaque-Specific Anti-HIV-1 Lentiviral Vector

To evaluate our hematopoietic stem and progenitor cell (HSPC)-based anti-HIV-1 gene therapy strategy in a non-human primate (NHP) model, we engineered a pigtail macaque (PM)-optimized lentiviral vector, designated PM-MNDU-anti-HIV-1-huEGFRt (Figure 1A). This construct was adapted from our previously described human-targeted “M1” vector, which demonstrated efficient hematopoietic reconstitution following transplantation of transduced human HSPCs into humanized BLT mice [20]. The PM-optimized multicistronic vector co-expresses three anti-HIV genes: an shRNA targeting the PM CCR5 co-receptor (PM-CCR5sh1005), an NHP-adapted CD4-based chimeric antigen receptor (D1D2CAR) containing 4-1BB and CD3ζ signaling domains, a membrane-anchored fusion inhibitor peptide (C46), and a non-functional truncated human EGFR surface marker (huEGFRt) utilized as a selectable tracking tag. The CD4-based D1D2CAR targets the HIV-1 envelope glycoprotein gp120, enabling recognition of HIV-1-infected cells.

To maximize silencing potency of CCR5sh1005, we introduced a single-nucleotide substitution (C8T) into the shRNA sequence to achieve a 100% sequence match with the PM CCR5 transcript. To validate this modification, we compared the knockdown efficiency of the human and PM-specific shRNAs in PM peripheral blood mononuclear cells (PBMCs). The PM-CCR5sh1005 vector mediated an approximate five-fold reduction in PM CCR5 expression within transduced huEGFRt^+^ cells, whereas the original human-targeted huCCR5sh1005 failed to induce significant knockdown (Figure 1B). Conversely, the human-specific shRNA successfully reduced human CCR5 expression in the human MT-4 CCR5 cell line, while the PM-specific shRNA did not, confirming stringent species specificity.

In addition to shRNA refinement, we codon-optimized the human CD4-based D1D2CAR sequence to align with the translational machinery preferences of NHPs. This optimization spanned the entire CAR construct, including the human CD4 extracellular domains (D1 and D2), the CD8-based hinge and transmembrane regions, and the 4-1BB and CD3ζ signaling domains. To match the native pigtail macaque sequence, we also introduced a single amino acid substitution (G216S) into the human 4-1BB co-stimulatory domain. Flow cytometric analysis confirmed robust multicistronic expression. Of the total vector-transduced cell population (43–49% overall transduction), 47–65% co-expressed both transgenes (e.g., 31.6% dual-positive out of 49.2% total transduced cells (Figure 1C).

### 3.2. Ex Vivo HSPC Gene Modification and Autologous Transplantation in Busulfan-Conditioned Pigtail Macaques

**Figure 2:**
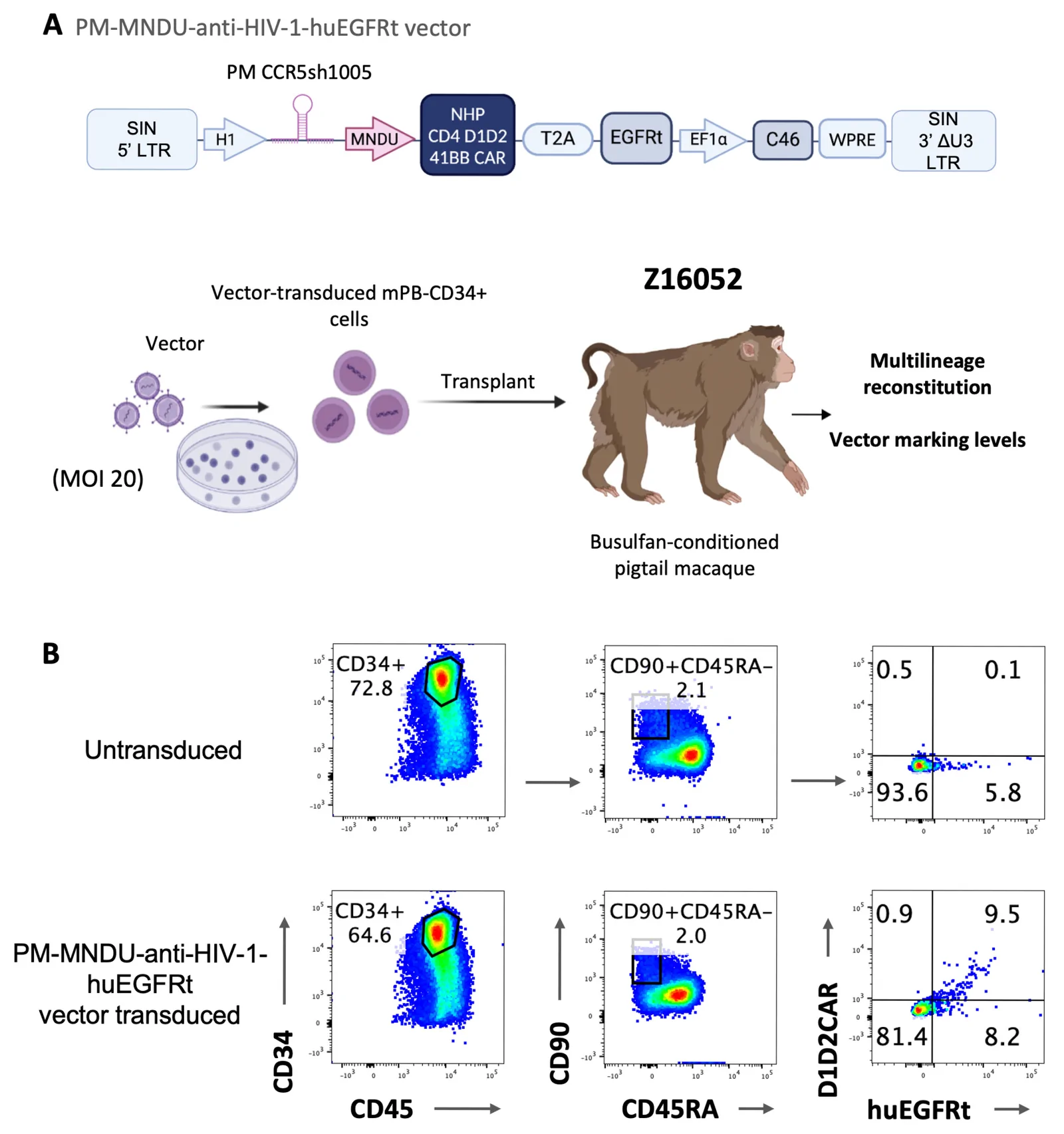
Single-vector autologous transplantation workflow in subject Z16052. (**A**) Autologous CD34^+^ HSPCs were mobilized into peripheral blood via recombinant human granulocyte colony-stimulating factor (G-CSF) and plerixafor (AMD3100). Mobilized CD34^+^ HSPCs were transduced ex vivo with the PM-MNDU-anti-HIV-1-huEGFRt vector at a multiplicity of infection (MOI) of 20. Following 1 day of culture, vector-modified cells were cryopreserved and later infused after myeloablative busulfan conditioning. (**B**) Quantification of D1D2CAR and huEGFRt cell-surface transgene expression in transduced long-term repopulating HSPCs (CD45^+^CD34^+^CD90^+^CD45RA^−^) ex vivo. Percent transgene expression levels were evaluated by monoclonal antibody (mAb) staining and flow cytometry at 4 days post-transduction. Note: C46 peptide surface expression could not be quantified in CD34^+^ HSPCs due to elevated background signal from anti-C46 mAb staining.

**Figure 3:**
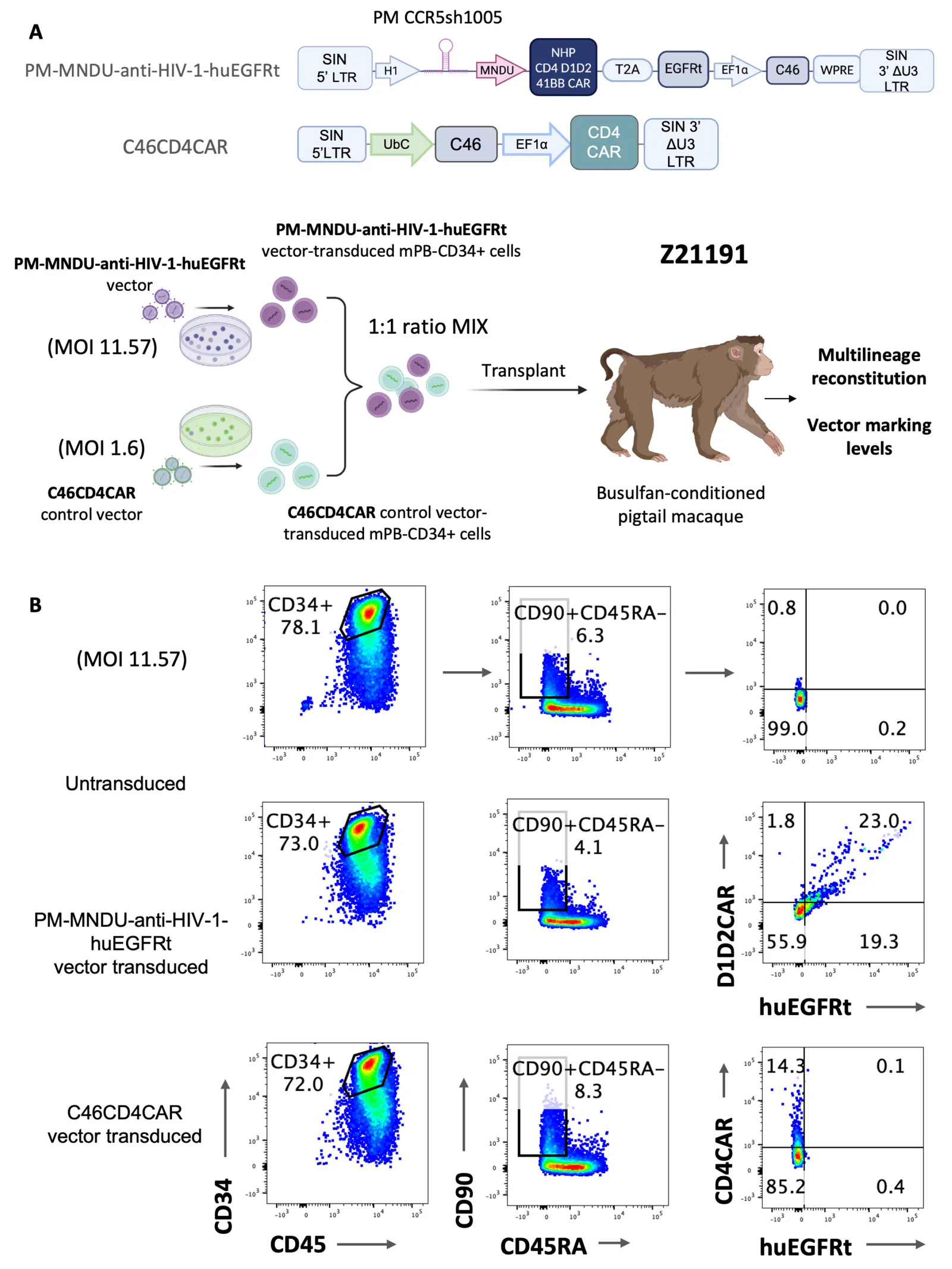
Dual-vector co-transplantation transplantation workflow in subject Z21191. (**A**) Autologous CD34^+^ HSPCs were mobilized as described above and divided into two fractions: equal portions were transduced with PM-MNDU-anti-HIV-1-huEGFRt (MOI 11.6) or control C46CD4CAR vector (MOI 1.6) [27]. Following 1 day of ex vivo culture, cells were pooled and cryopreserved. On the day of infusion, pooled cells were transplanted into Z21191 following identical busulfan conditioning. (**B**) Quantification of D1D2CAR (or control CD4CAR) and huEGFRt cell-surface transgene expression in transduced long-term repopulating HSPCs (CD45^+^CD34^+^CD90^+^CD45RA^−^) ex vivo. Percent transgene expression levels were evaluated by monoclonal antibody (mAb) staining and flow cytometry at 4 days post-transduction. Note: C46 peptide surface expression could not be quantified in CD34^+^ HSPCs due to elevated background signal from anti-C46 mAb staining.

To evaluate the in vivo engraftment potential of HSPCs transduced with the PM-specific vector, we performed autologous HSPC transplants in two juvenile pigtail macaques (Z16052, female; Z21191, male) using distinct experimental designs (Figure 2A and Figure 3A). An untransplanted, non-SHIV-challenged pigtail macaque was maintained in parallel as an uninfected control.

#### 3.2.1. Cohort 1: Single-Vector Evaluation (Subject Z16052)

In our first subject (Z16052), autologous HSPCs were mobilized into the peripheral blood using G-CSF and plerixafor (AMD3100) and subsequently harvested (Figure 2A). Mobilized HSPCs were transduced ex vivo with the PM-MNDU-anti-HIV-1-huEGFRt vector at an MOI of 20. Following one day of ex vivo culture, vector-modified cells were cryopreserved and later infused into Z16052 following pre-conditioning with a myeloablative busulfan regimen.

We evaluated transduction efficiency using a subset of cells maintained in *ex vivo* culture to minimize the effect of pseudotransduction and evaluated expression of D1D2CAR and huEGFRt specifically within the CD45^+^CD34^+^CD90^+^CD45RA^−^ subpopulation, which is highly enriched for long-term repopulating HSPCs principally responsible for durable, multilineage hematopoietic reconstitution [46]. At day 4 post-transduction, the D1D2CAR^+^huEGFRt^+^ co-expressing cell population reached 10.4% within this long-term HSPC fraction (Figure 2B). Within total CD34^+^ cells, D1D2CAR^+^huEGFRt^+^ expression reached 12.4% (Figure S1).

#### 3.2.2. Cohort 2: Dual-Vector Co-Transplantation Strategy (Subject Z21191)

For our second subject (Z21191), we refined the experimental protocol to incorporate an internal vector control (Figure 3A). While single-vector transplantation validates overall feasibility, it cannot easily decouple vector-specific in vivo behavior from host-specific biological variation. To directly control for and precisely measure the in vivo engraftment, persistence, and dynamics of the PM-MNDU-anti-HIV-1-huEGFRt vector, we utilized a dual-vector co-transplantation strategy.

As an internal control, we selected C46CD4CAR, a previously validated lentiviral vector expressing a CD4-based CAR and the C46 fusion inhibitor in pigtail macaque HSPC transplantation models. C46CD4CAR expresses the full-length CD4-based CAR (D1-D4 domains) and lacks the 4-1BB co-stimulatory domain. This vector has previously been demonstrated to efficiently transduce pigtail macaque HSPCs and mediate long-term multilineage engraftment [27]. By separately transducing portions of the same starting HSPC pool with either the experimental PM-MNDU-anti-HIV-1-huEGFRt vector or the control C46CD4CAR vector and mixing them prior to infusion, we could monitor the distinct cell populations within identical in vivo microenvironments. Because the two vectors possess distinct genetic sequences, their respective in vivo DNA marking levels were quantitatively distinguished and tracked over time via vector-specific digital PCR (dPCR).

To minimize the duration of ex vivo culture before transplant, vector-modified cells were combined and cryopreserved one day after transduction. A subset of cells were maintained in culture *ex vivo* to evaluate transduction efficiency. At day 4 post-transduction, the D1D2CAR^+^huEGFRt^+^ co-expressing cell population in the PM-MNDU-anti-HIV-1-huEGFRt vector-transduced cells reached 23% within the CD90^+^CD45RA^−^ long-term HSPC fraction (Figure 3B). A total of 14.3% of the C46CD4CAR vector-transduced cells expressed full-length CD4CAR (Figure 3B). Within total CD34^+^ cells, D1D2CAR^+^huEGFRt^+^ and CD4CAR^+^ expression reached 14.3% and 4.4%, respectively (Figure S1).

A summary of animal characteristics and graft composition at transplantation for both subjects is presented in Table 1. The pre-infusion vector copy number (VCN) in HSPC products were comparable in subject Z21191 (0.9 copies/cell for PM-MNDU-anti-HIV-1-huEGFRt vs. 0.7 copies/cell for C46CD4CAR), despite being transduced at a 7.25-fold difference in MOI. Subject Z16052 received an HSPC product with a higher pre-infusion VCN 5.2 copies/cell. Notably, subject Z16052 received approximately five-fold fewer long-term repopulating CD45^+^CD34^+^CD90^+^CD45RA^−^ HSPCs than Z21191 (1.74 × 10^5^ cells/kg vs. 8.84–9.86 × 10^5^ cells/kg).

### 3.3. Post-Transplant Reconstitution, Engraftment Dynamics, and Safety

Following myeloablative busulfan conditioning and HSPC transplantation, both subjects exhibited similar busulfan plasma pharmacokinetics, with drug levels steadily declining with an elimination half-life of t_1/2_ ≈ 3h (Figure S2A). Both animals demonstrated standard recovery kinetics for neutrophils, platelets, and peripheral blood lymphocytes, consistent with historical observations (Figure S2B). Relative frequencies of major peripheral blood hematopoietic lineages prior to transplantation and at multiple timepoints post-transplant demonstrate normal multilineage hematopoietic reconstitution (Figure S3). No significant hepatic or renal toxicities were observed, as assessed by normal serum chemistry profiles (blood urea nitrogen, creatinine, alkaline phosphatase, ALT, AST, and GGT; Figure S4A,B).

**Figure 4:**
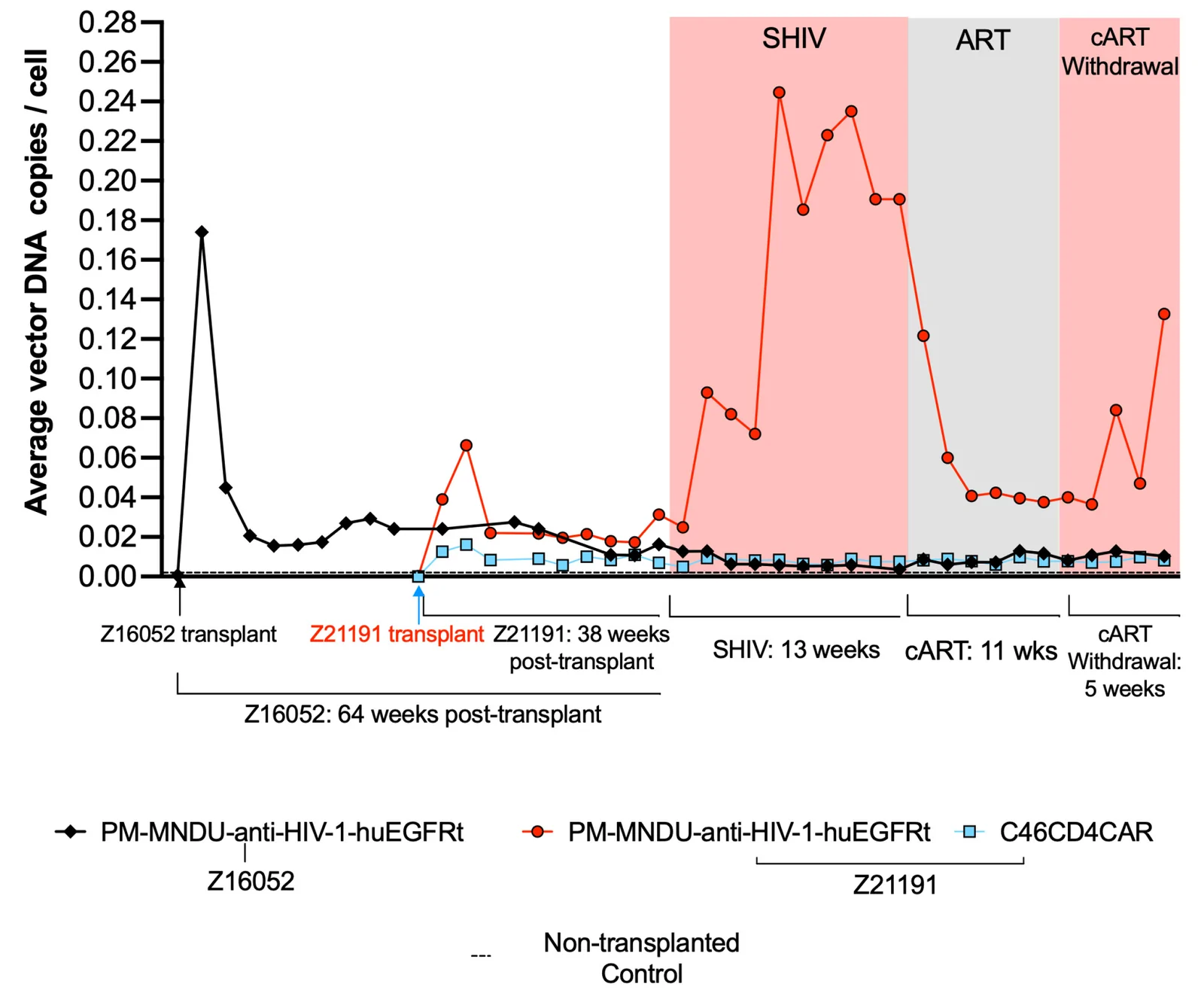
Longitudinal vector DNA marking kinetics of PM-MNDU-anti-HIV-1-huEGFRt and control C46CD4CAR in peripheral blood. Average vector DNA copy numbers per cell were quantified in peripheral blood mononuclear cells (PBMCs) from subjects Z16052 and Z21191 using sequence-specific digital PCR (dPCR) primer–probe sets across the longitudinal in vivo study timeline. PBMCs isolated from an untransplanted, uninfected pigtail macaque served as a negative control.

We next quantified in vivo vector DNA marking in peripheral blood mononuclear cells (PBMCs) collected longitudinally post-transplantation. Because surface expression levels of huEGFRt and CD4-based CAR were initially below the threshold of detection by flow cytometry, vector DNA marking was quantified via genomic dPCR using vector-specific primer–probe sets. Peak vector DNA marking occurred shortly after transplantation, reaching 0.18 copies/cell at week 1 for Z16052 and 0.07 copies/cell at week 2 for Z21191 (Figure 4). After 4 weeks post-transplant, gene marking levels stabilized at approximately 0.02 vector DNA copies/cell for 64 weeks (Z16052) and 38 weeks (Z21191) prior to SHIV challenge (Figure 4). In Z21191, average control C46CD4CAR vector copies stabilized at 0.01 copies/cell starting at week 1 post-transplant (Figure 4). Compared to historical benchmark studies investigating the C46CD4CAR vector in total body irradiation (TBI)- or busulfan-conditioned rhesus or pigtail macaques, marking levels of 0.01–0.02 copies/cell fall within the anticipated physiological range for reduced-intensity busulfan-conditioned transplants [27,47].

Collectively, these data demonstrate that busulfan conditioning supports safe hematopoietic recovery and stable engraftment of PM-MNDU-anti-HIV-1-huEGFRt vector-transduced autologous HSPCs, resulting in long-term persistence and stable integration of vector-marked progeny in two pigtail macaques.

### 3.4. Multilineage Reconstitution and Expansion of Vector-Modified Cells Following SHIV Infection and cART Interruption

To investigate whether vector-modified cells undergo positive selection and expansion in response to active viral replication, both animals were challenged intravenously with 16700 TCID_50_ of SHIV-1157-ipd3N4 following the establishment of stable engraftment. Longitudinal analysis revealed distinct vector marking dynamics between the two subjects (Figure 4). In Z16052, average PM-MNDU-anti-HIV-1-huEGFRt vector DNA copy numbers remained stable post-infection. In contrast, Z21191 exhibited a progressive, 12-fold increase in PM-MNDU-anti-HIV-1-huEGFRt vector marking, peaking at 0.24 copies/cell by 8 weeks post-infection, consistent with selective expansion of the PM-MNDU-anti-HIV-1-huEGFRt population upon viral challenge (Figure 4). Control C46CD4CAR vector marking levels remained stable during this same timeframe.

**Figure 5:**
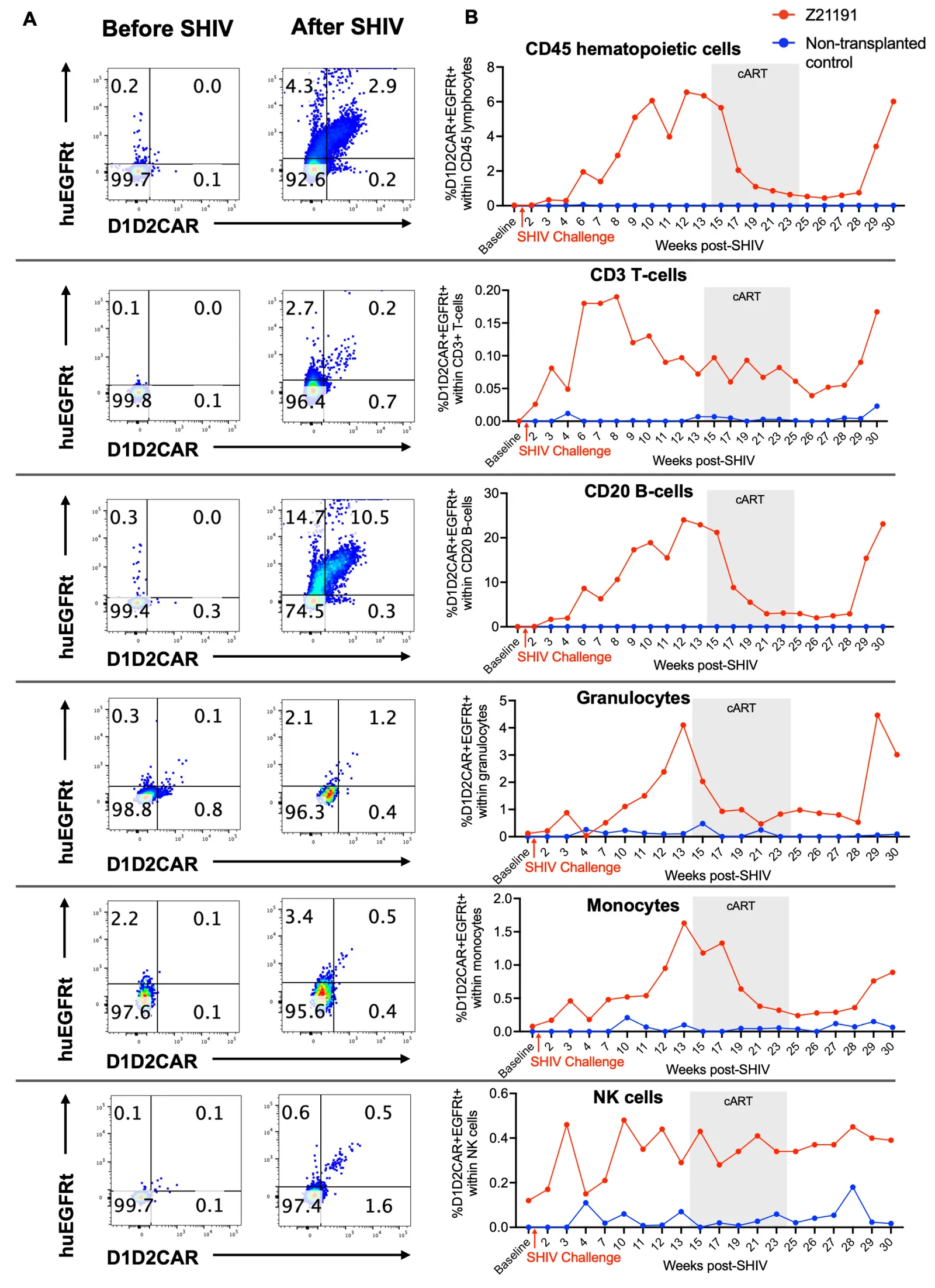
Multilineage expansion, cART suppression, and post-treatment rebound of gene-modified cells in subject Z21191. (**A**) Representative flow cytometry plots illustrating the increase in D1D2CAR^+^huEGFRt^+^ double-positive cells across major peripheral blood hematopoietic lineages pre- and post-SHIV challenge (8–10 weeks post-challenge). (**B**) Longitudinal frequency kinetics of D1D2CAR^+^huEGFRt^+^ cells within peripheral blood subsets before infection, during acute SHIV infection, under suppressive cART, and following cART withdrawal. Lineage gating includes total leukocytes (CD45^+^), T cells (CD3^+^), B cells (CD20^+^), monocytes (CD11b^+^CD14^high^), NK cells (NKG2a^+^), and granulocytes (CD11b^+^CD14^low^). Cell-surface transgene expression was determined by monoclonal antibody (mAb) staining and flow cytometry in PBMCs isolated from subject Z21191. PBMCs from an untransplanted, uninfected macaque served as a negative gating control.

To determine whether this expansion spanned multiple lineages, we determined the frequency of D1D2CAR^+^huEGFRt^+^ cells within peripheral blood subsets by flow cytometry (Figure 5). During this period, D1D2CAR^+^huEGFRt^+^ cells expanded across both lymphoid and myeloid compartments in CD45^+^ PBMCs: total leukocytes (CD45^+^) exhibited a 19-fold increase; CD3^+^ T cells, a 3.7-fold increase; CD20^+^ B cells, a 14-fold increase; CD11b^+^CD14^high^ monocytes, a 3.5-fold increase; NKG2a^+^ NK cells, a 2.6-fold increase; and CD11b^+^CD14^low^ granulocytes, a 2.7-fold increase.

Unexpectedly, D1D2CAR^+^huEGFRt^+^ co-expression within CD3^+^ T cells declined prior to cART initiation (Figure 5 and Figure S5). Concurrently, expression of the immune checkpoint marker PD-1 displayed a two-fold increase on CAR^+^ T cells, pointing toward cell-mediated exhaustion driven by high viral loads during primary SHIV infection (Figure S5). To confirm that active SHIV replication drove this expansion, Z21191 was administered an 11-week regimen of combination antiretroviral therapy (cART). Initiation of cART resulted in a 4.75-fold decrease in the vector DNA copy number in peripheral blood mononuclear cells, with low-level residual vector marking persisting throughout the suppression period (Figure 4 and Figure 5). Following cART withdrawal, viral reactivation induced a rapid secondary expansion of vector-modified cells, mirroring an anamnestic response to renewed viral antigen exposure (Figure 4 and Figure 5).

### 3.5. Cetuximab-Mediated Negative Selection via the huEGFRt Safety Switch

The inclusion of huEGFRt in our multicistronic vector could serve as a safety feature, allowing Cetuximab (CTX; an anti-EGFR monoclonal antibody)-mediated ablation of transgene-expressing cells if adverse events arise, as previously shown in NSG and humanized BLT mice [20,39,40]. Following cART withdrawal and the subsequent rebound of D1D2CAR^+^huEGFRt^+^ cells in Z21191, we evaluated the efficacy of CTX-mediated negative selection in vivo.

**Figure 6:**
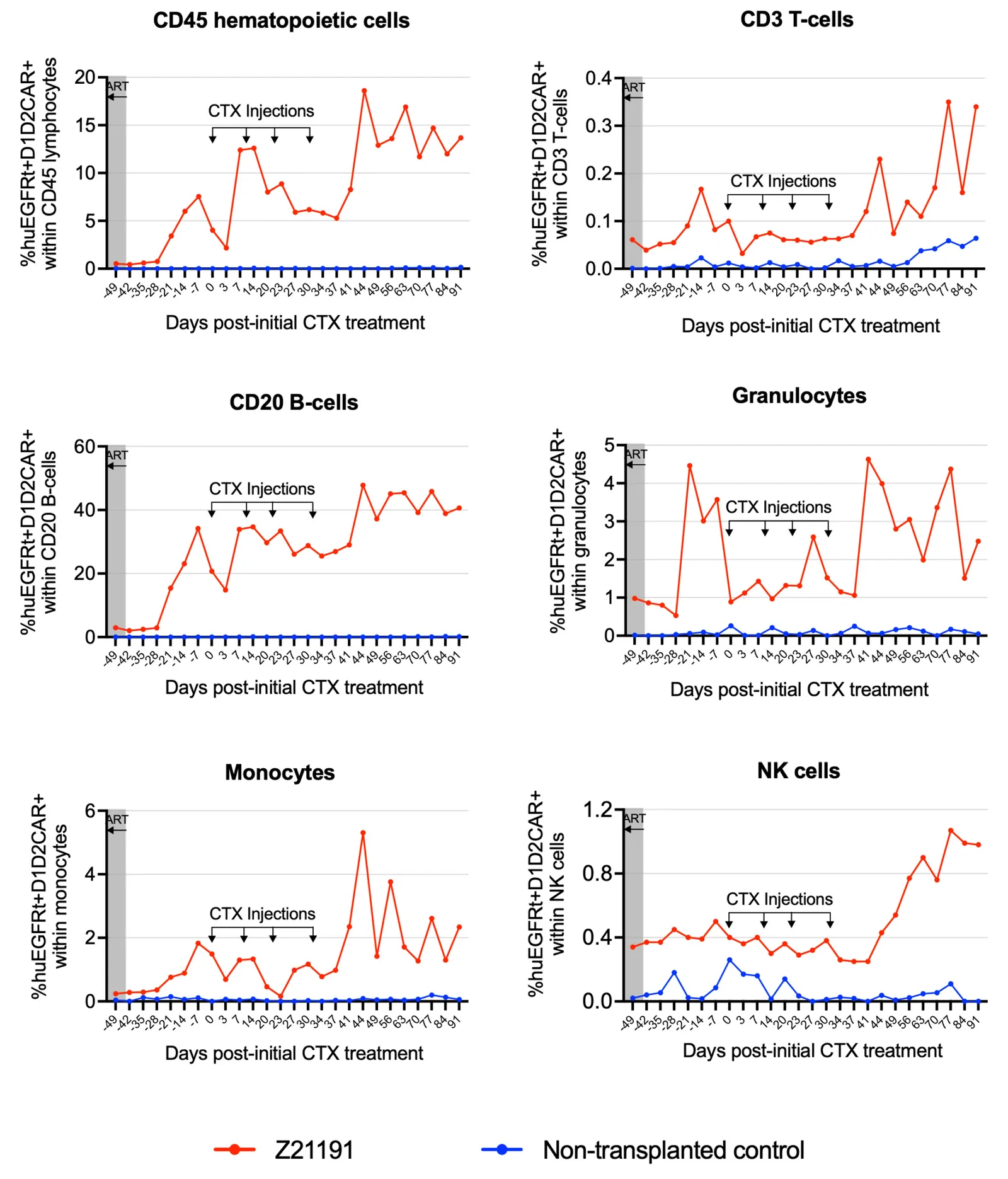
Cetuximab (CTX)-mediated negative selection of transgene-expressing cells in vivo. Cetuximab administration induced a transient, multilineage reduction in the frequency of D1D2CAR^+^huEGFRt^+^ double-positive cells in peripheral blood from subject Z21191. Transgene-expressing cell frequencies surged following cART withdrawal, were blunted/reduced during active CTX infusion, and subsequently rebounded following CTX discontinuation. Cell-surface co-expression of huEGFRt and D1D2CAR across multilineage subsets was quantified by monoclonal antibody staining and flow cytometry. PBMCs from an untransplanted, uninfected macaque served as a negative gating control.

An initial dose of CTX (4 mg/kg IV) resulted in a minimal, transient reduction in % D1D2CAR^+^huEGFRt^+^ cells (Figure 6). Because rapid ongoing cell expansion post-cART interruption likely masked early ablation kinetics, Z21191 was administered three additional doses of CTX (each spaced 1–2 weeks apart), culminating in a final dose of 5 mg/kg. CTX treatment blunted the expansion phase, stabilizing D1D2CAR^+^huEGFRt^+^ expression between days 7 and 41 post-CTX initiation (Figure 6 and Figure S6). Analysis of vector DNA marking confirmed a transient reduction in average PM-MNDU-anti-HIV-1-huEGFRt vector DNA copies following the third and fourth CTX administrations (Figure S7).

Upon CTX withdrawal, multilineage D1D2CAR^+^huEGFRt^+^ populations expanded back to levels comparable to those observed during primary SHIV infection, with vector DNA marking rising above 0.18 copies/cell until study completion (Figure 6 and Figure S7). Thus, huEGFRt functions as a biologically active tag capable of transiently reducing vector-modified cells in vivo. The four-dose CTX regimen was well-tolerated, with no observed systemic toxicity or significant weight loss in Z21191.

### 3.6. Plasma Viral Load Kinetics Across Therapeutic Interventions

**Figure 7:**
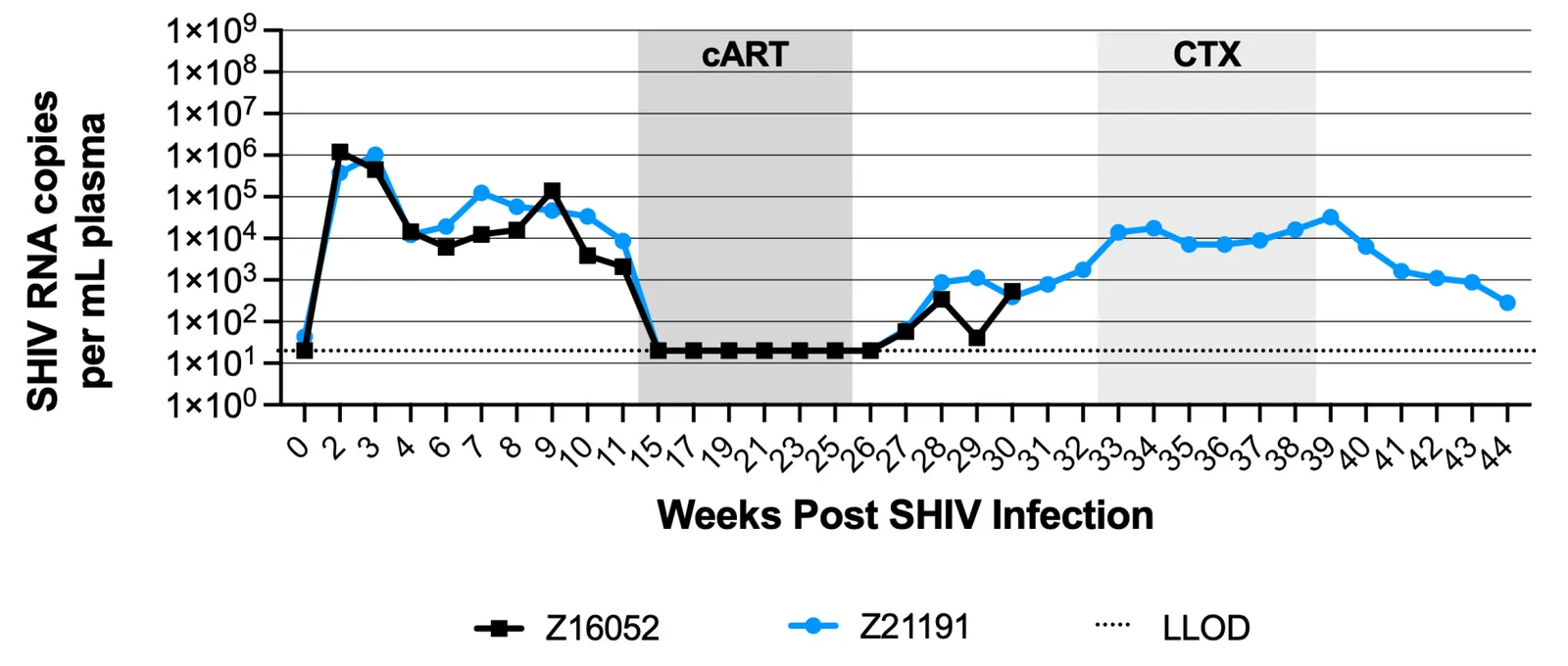
Longitudinal plasma viral load kinetics across therapeutic interventions in SHIV-challenged macaques. Acute SHIV viremia peaked at 10^6^ viral RNA copies/mL within 0–3 weeks post-challenge in both subjects before establishing a persistent infection set-point. Combination antiretroviral therapy (cART) mediated robust viral suppression, whereas cART interruption prompted rapid viral rebound. In subject Z21191, Cetuximab (CTX)-mediated negative selection of transgene-expressing cells was accompanied by a transient ∼1-log elevation in plasma viremia, which subsequently declined following CTX withdrawal upon the secondary expansion of gene-modified cells. Plasma SHIV RNA copy numbers were quantified by quantitative reverse transcription PCR (qRT-PCR). The horizontal dashed line denotes the assay’s lower limit of detection (LLOD).

Plasma SHIV RNA kinetics reflected the functional interplay between vector-modified cells, viral replication, cART, and CTX administration (Figure 7). Following SHIV-1157-ipd3N4 challenge, acute plasma viremia peaked at 10^6^ SHIV RNA copies/mL within 3 weeks post-infection in both animals, stabilizing into a persistent infection (10^4^–10^5^ copies/mL) between weeks 4 and 11 (Figure 7).

In Z21191, stabilization of set-point viral load directly coincided with the selective expansion of PM-MNDU-anti-HIV-1-huEGFRt vector-modified cells (Figure 4 and Figure 7). As expected, cART administration suppressed viral load to undetectable levels (<20 copies/mL) in both subjects, and cART cessation prompted rapid viral rebound (Figure 7). Subject Z16052 reached its scheduled study endpoint shortly post-cART due to the non-responsiveness of vector DNA copy numbers after SHIV challenge. Viral suppression by cART mirrored the results observed in our previous pigtail macaque study of the C46CD4CAR control vector (Figure S8). In the study, one of two C46CD4CAR-modified HSPC-transplanted pigtail macaques (CAR2) showed reduced viral rebound compared to the control group after cART suppression [27].

During the CTX treatment phase in Z21191, average viral load increased by 1 − log (∼1.48 × 10^4^ SHIV RNA copies/mL during weeks 33–39) compared to pre-treatment levels (∼9.93 × 10^2^ copies/mL during weeks 28–32) (Figure 7). This transient increase in viral load inversely correlated with the partial depletion of PM-MNDU-anti-HIV-1-huEGFRt vector-modified cells by CTX. Following CTX withdrawal, viral load subsequently declined to 10^3^ copies/mL by study termination, mirroring the secondary recovery and expansion of vector-modified effector cells (Figure 7 and Figure S8).

### 3.7. Multilineage Reconstitution and Tissue Distribution in Secondary Lymphoid Organs

In pre-infection tissue biopsies from Z21191, PM-MNDU-anti-HIV-1-huEGFRt and C46CD4CAR vector-marked cells were detected in the spleen, mesenteric lymph nodes, and axillary lymph nodes at levels comparable to baseline PBMCs (0.02 and 0.005 copies/cell, respectively; Figure S9). D1D2CAR-expressing cells were also distributed in multiple hematopoietic lineages (CD4 T-cells, CD8 T-cells, CD20^+^ B-cells, monocytes, and NK cells) in the tissues (Figure S10). At necropsy post-SHIV infection, PM-MNDU-anti-HIV-1-huEGFRt vector marking increased five-fold in the spleen and two-fold in the mesenteric and axillary lymph nodes (Figure S9). Control C46CD4CAR vector levels increased in the mesenteric lymph nodes, but not in the spleen or axillary nodes (Figure S9). Extended tissue analysis at necropsy demonstrated widespread distribution of vector-modified cells across tissues harboring cells with SHIV proviral DNA. (Figures S11 and S12). In the bone marrow, both PM-MNDU-anti-HIV-1-huEGFRt and C46CD4CAR vectors were detected in enriched CD34^+^CD90^+^ HSPCs (0.025 and 0.01 DNA copies/cell, respectively), confirming persistent gene marking within long-term stem cell compartments. In the thymus, vector-marked cells were detected in thymic tissue. In the peripheral lymph nodes, marked cells were detected in iliac, submandibular, and inguinal lymph nodes (Figure S11). Together, these results confirm that HSPC-derived anti-HIV/SHIV effector progeny broadly traffic to and persist within primary and secondary lymphoid tissues, positioning them at key sites of viral persistence and replication in pigtail macaques.

## 4. Discussion

In this study, we evaluated the feasibility, safety, and dynamics of an anti-HIV-1 hematopoietic stem and progenitor cell (HSPC) gene therapy strategy in a large animal model, the pigtail macaque (*Macaca nemestrina*). We developed a species-optimized lentiviral vector, PM-MNDU-anti-HIV-1-huEGFRt, expressing multi-pronged anti-HIV genes comprising a functional shRNA against PM CCR5, a C46 fusion inhibitor, a CD4-based CAR (D1D2CAR 4-1BB), and a huEGFRt safety tracking marker. Our findings demonstrate that species-optimized lentiviral transduction enables stable, long-term engraftment of autologous HSPCs in busulfan-conditioned macaques without associated regimen-related toxicity. Vector-modified progeny successfully populated peripheral blood and lymphoid tissues, exhibited antigen-driven multilineage expansion in response to SHIV challenge, and demonstrated sensitivity to Cetuximab (CTX)-mediated negative selection in vivo.

Our study utilized reduced-intensity busulfan conditioning. Total body irradiation (TBI)-based regimens carry severe toxicities and are unsuitable for otherwise healthy people living with HIV receiving suppressive antiretroviral therapy. Busulfan represents a more clinically translatable agent and serves as the standard conditioning drug for autologous HSPC gene therapy trials in humans [48,49]. Although baseline vector marking stabilized at modest levels (0.01–0.02 copies/cell)—lower than historical benchmarks following myeloablative TBI—these gene-modified cells persisted long-term and underwent substantial expansion following viral challenge. Plasma viral load showed similar trends of viral load rebound between our busulfan-conditioned pigtail macaque that demonstrated gene-modified cell expansion compared with a previously studied CD4-CAR-expressing pigtail macaque that received myeloablative TBI (Figure S8).

The divergent response kinetics between the two subjects in this study highlight important biological variables in large animal HSPC gene therapy. While both animals achieved stable baseline engraftment, robust antigen-driven expansion following SHIV challenge was observed only in subject Z21191. This degree of variability aligns with the aforementioned study using the C46CD4CAR construct, where one of two animals sustained higher gene marking [27]. Differences in sex, grafted stem cell composition, and transduction efficiency within specific progenitor subsets may have contributed to these distinct in vivo trajectories [47]. A notable variable was the graft composition: subject Z16052 received approximately five-fold fewer long-term repopulating CD34^+^CD90^+^CD45RA^−^ HSPCs than Z21191 (1.74 × 10^5^ cells/kg vs. 8.84–9.86 × 10^5^ cells/kg; Table 1). Given the small cohort size, these observations serve as important hypothesis-generating insights regarding graft quality and engraftment thresholds.

In our previous huBLT mouse study, our multifunctional strategy demonstrated anti-HIV efficacy that can be attributed to relatively high transduction efficiency of human fetal liver-derived CD34^+^ HSPC, engraftment levels, and multilineage reconstitution including T-cells. This was supported with lower HIV-infected target cell kinetics, likely lowering the threshold required to observe viral suppression. In this present study, we faced several challenges to evaluate all three anti-HIV component and their anti-viral efficacy in the pigtail macaque model. Consistent with previous studies utilizing anti-HIV CAR, transgene-marked cells in Z21191 consisted of mostly B-cells with a much smaller population within T-cells in peripheral blood and lymphoid tissues [27]. The low percentage of transgene-marked T-cells can be attributed to a relatively low level of engraftment and sub-optimal T-cell reconstitution in busulfan-conditioned immunocompetent pigtail macaques. As a result, robust flow cytometric detection of certain elements in T cells was precluded. Native CCR5 expression in macaque T cells is typically low (<5–10%) and C46 detection by flow cytometry suffered from high non-specific background [50,51]. Together, these technical hurdles and the small cohort of animals restrict definitive functional demonstration of the anti-HIV components in pigtail macaque. Improvement of T-cell reconstitution and large-scale animal cohorts would parse these individual components including C46 and CD4-CAR.

C46 and CD4-CAR components can function via a tropism-independent mechanism, ensuring sustained antiviral pressure against both R5- and X4-strains. This is particularly relevant because the HIV-1 population in patients may include X4 or dual-tropic variants, and selective pressure can favor outgrowth of those populations. Since SHIV is R5-tropic, our present model primarily evaluates viral response against R5-tropic virus and does not directly address the activities against X4 or dual-tropic variants. Studies incorporating viruses with alternative tropism will be valuable to evaluate the breadth of our multi-tropism approach.

The integration of an inducible negative selection marker represents an essential translational safeguard against potential insertional oncogenesis or uncontrolled immune effector CAR T cell proliferation [52,53,54,55,56]. HSPC gene therapy poses theoretical safety risks, including malignant transformation. Anti-HIV-1 CAR-T vector-modified cells may potentially cause unexpected health issues such as cytokine release syndrome and CAR-T cell-related encephalopathy syndrome in hosts, as seen in cancer immunotherapy. We selected huEGFRt because its compact cDNA sequence is accommodated within multicistronic vectors, and it provides a dual function as both a non-immunogenic cell-surface tag for flow cytometric tracking and a target for antibody-mediated ablation via Cetuximab (CTX), an FDA-approved chimeric IgG1 monoclonal antibody.

Upon CTX administration in subject Z21191, we observed a stabilization of the expansion phase and a partial, transient reduction in vector-marked cells. The partial and transient depletion was followed by a post-withdrawal rebound of vector-marked cells. Complete ablation was likely constrained by continuous production of huEGFRt^+^ cells from transplanted vector-modified HSPCs, rapid ongoing post-cART rebound kinetics, and antibody distribution dynamics. Currently, huEGFRt serves only as a partial safety mechanism in the pigtail macaque model which may be insufficient as a definitive safety mechanism for an HSPC-based gene therapy if long-term repopulating cells can persist and regenerate adverse progenies. In our huBLT mice study, human NK cell supplementation and cytokine (IL-15)-expressing vector were required to achieve robust ADCC. Perhaps similar therapeutic enhancement strategies (expressing IL-15) can improve efficacy in non-human primates. Conventional EGFRt/CTX systems have been reported to mediate slow and incomplete depletion, with limited reduction in vector-marked cells in the bone marrow, prompting efforts to improve EGFRt expression through receptor design and antibody-mediated clearance [57]. Optimizing CTX dosing schedules or combining it with additional complementary safety kill switches will also be important avenues for refining this safety feature to achieve additive or synergistic clearance.

In our humanized BLT mouse study, MNDU3 demonstrated the highest potency for robust, multi-transgene co-expression. Therefore, our species-optimized lentiviral vector utilizes the MNDU3 promoter to co-express both D1D2CAR and huEGFRt. However, we acknowledge that this strong internal promoter carries genotoxic risks associated with vector-marked cell outgrowth and potential clonal expansion. A previous gene therapy trial for Cerebral Adenoleukodystrophy (eli-cel; Lenti-D) consisting of autologous CD34^+^ cells transduced with MNDU3-lentiviral vector have resulted in cases of hematological cancer associated with clonal vector insertions within oncogenes [58]. The genomic analyses suggest vector integration alone was insufficient for malignant transformation and that additional somatic alternation and clonal evolution contributed to disease progression. However, another study highlighted that strong constitutive promoters can lead to abnormal clonal hematopoiesis in the rhesus macaque model that warrants careful selection and characterization of promoters [55]. Because our CTX-mediated huEGFRt negative selection marker is only partially functional in the pigtail macaque model, it might be of best interest for safety to transition to safer promoters for CARs (e.g., lineage-specific or self-inactivating internal promoters) in addition to combining huEGFRt with complementary, synergistic negative selection strategies to mitigate long-term genotoxicity risks.

## 5. Conclusions

In conclusion, this study demonstrates the feasibility and safety of a multifunctional “defend and attack” HSPC gene therapy strategy in the busulfan-conditioned pigtail macaque model. Autologous transplantation and engraftment of vector-modified HSPCs resulted in sustained multilineage vector marking across peripheral blood and secondary lymphoid tissues without adverse affects. In one animal, upon SHIV challenge, vector-modified cells underwent robust antigen-driven multilineage expansion, cART-induced viral suppression and rapid rebound following cART interruption. Administration of CTX demonstrated partial, transient reduction in huEGFRt^+^ cells without adverse effects. These findings supports the feasibility of evaluating a multi-pronged anti-HIV HSPC gene therapy strategy with a negative selection marker in a non-human primate model.

## Figures and Tables

**Table 1:** Pre-infusion characteristics of transduced CD34^+^ HSPC products.

Animal	Vector	VCN ^1^ (MOI)	Total Cells	%CD34^+^	%CD90^+^	Total CD34^+^	Total CD90^+^	CD34/kg	CD90/kg
Z16052 Female 6.3 kg *	PM-MNDU	5.20 (20)	5.60 × 10^7^	87%	2%	4.87 × 10^7^	1.18 × 10^6^	7.22 × 10^6^	1.74 × 10^5^
Z21191 Male 4 kg *	PM-MNDU	0.94 (11.6)	1.42 × 10^7^	94%	25%	1.34 × 10^7^	3.54 × 10^6^	3.34 × 10^6^	8.84 × 10^5^
	C46CD4CAR	0.70 (1.6)	1.73 × 10^7^	94%	23%	1.62 × 10^7^	3.94 × 10^6^	4.06 × 10^6^	9.86 × 10^5^

Table shows percentage and cell counts of the infusion products that were cryopreserved at Day 1 post-transduction. Abbreviations. PM-MNDU represents PM-MNDU-anti-HIV-1-huEGFRt vector. * Weight at transplantation (kg). ^1^ Pre-infusion vector DNA copy number (VCN) was determined by dPCR on Day 6 post-transduction following ex vivo culture to allow decay of unintegrated vector DNA.

## Data Availability

The datasets generated and analyzed during the current study are available from the corresponding author upon reasonable request.

## Supplementary Materials

The following supporting information can be downloaded at https://www.mdpi.com/article/10.3390/v18101133/s1, Figure S1: Quantification of D1D2CAR, CD4CAR, and huEGFRt cell-surface transgene expression in total CD34^+^ HSPCs ex vivo; Figure S2: Busulfan pharmacokinetics and post-transplant hematopoietic recovery kinetics in subjects Z16052 and Z21191; Figure S3: Multilineage hematopoietic cell reconstitution; Figure S4: Assessment of renal and hepatic toxicity via longitudinal serum chemistry profiles in busulfan-conditioned pigtail macaques; Figure S5: Dynamics of T-cell exhaustion marker PD-1 on D1D2CAR^+^huEGFRt^+^ T cells post-SHIV challenge; Figure S6: Change in the frequency of huEGFRt^+^D1D2CAR^+^ cells across multilineage cells; Figure S7: Vector DNA marking dynamics in peripheral blood during Cetuximab (CTX)-mediated negative selection in subject Z21191; Figure S8: Plasma viral loads of C46CD4CAR vector (CAR1 and 2) or a no-HIV-1 gene vector (Control 1 and 2)-modified HSPC-transplanted pigtail macaques from a previously published study; Figure S9: Engraftment and expansion of vector-marked cells across secondary lymphoid tissues in subject Z21191; Figure S10: Surface phenotyping of D1D2CAR-expressing cells in PBMC and tissues of Z21191; Figure S11: Biodistribution of vector-modified HSPCs and immune cells in bone marrow, thymus, and peripheral lymph nodes of subject Z21191 at necropsy; Figure S12: Quantification of SHIV proviral DNA in peripheral blood and tissues.
