# Supplementary material for: Evaluation of Anti-HIV-1 HSPC-Based Gene Therapy with Negative Selection Marker in Pigtail Macaques
Source: Viruses. 2026 Oct 13;18(10):1133. doi: 10.3390/v18101133 (PMC13709364; doi:10.3390/v18101133)
Supplement: Supplementary file 1 [file viruses-18-01133-s001.zip › Supplemental Figures.pdf]

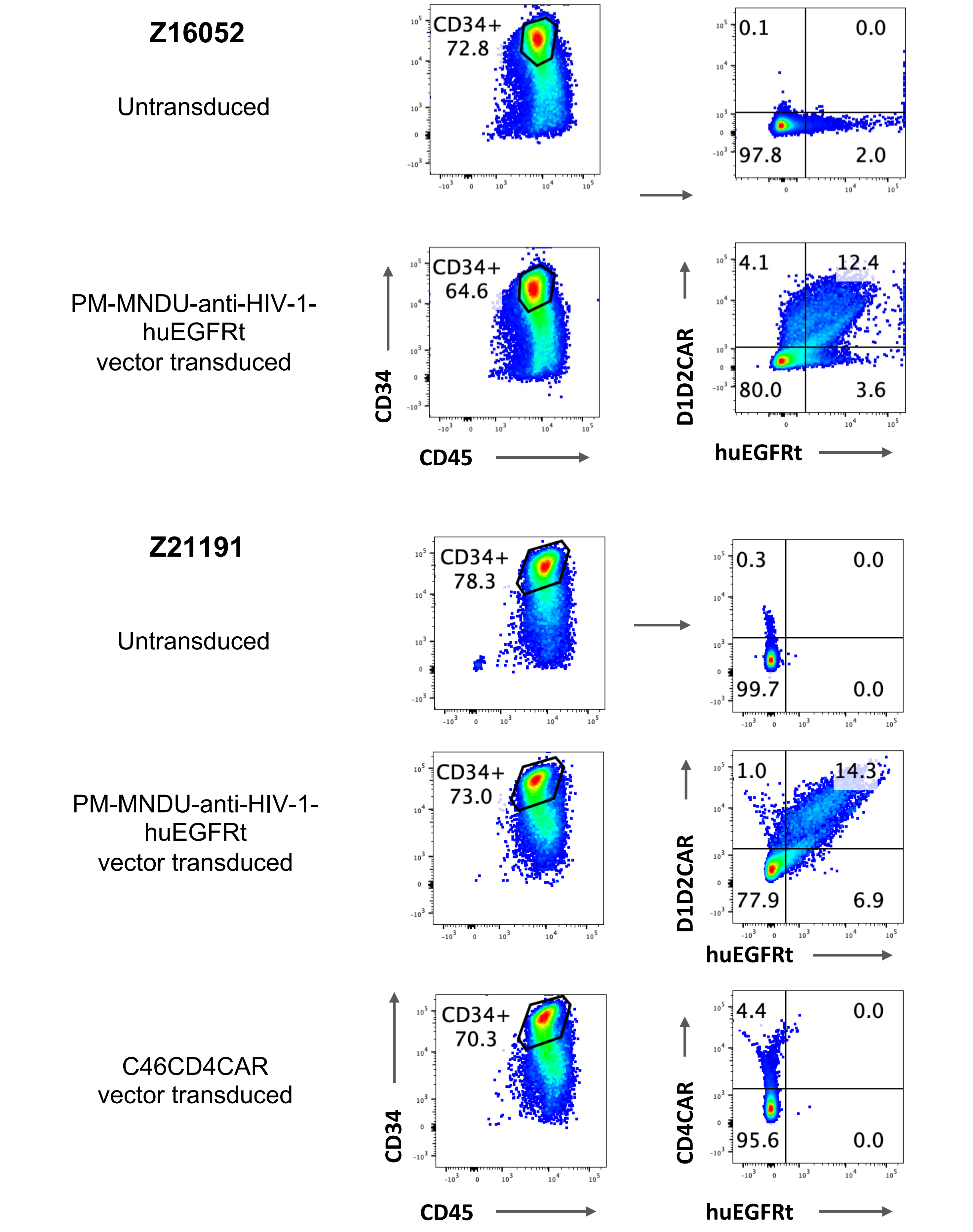

Figure S1. Quantification of D1D2CAR, CD4CAR and huEGFRt cell-surface transgene expression in total CD34+ HSPCs ex vivo. Transgene expression levels were evaluated by monoclonal antibody staining and flow cytometry at 4 days post-transduction.

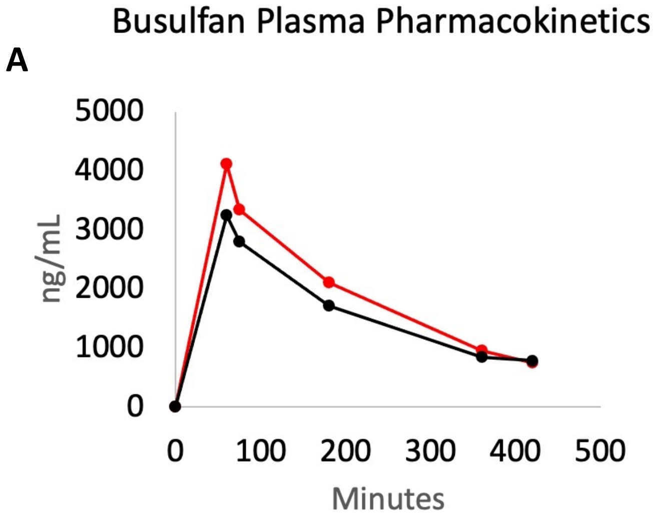

Animal	Dose (mg/kg/day x4)	Measured AUC (uMol*min)
Z16052	3.5	3251 (Dose 2)
Z21191	3.5	3350 (Dose 1)

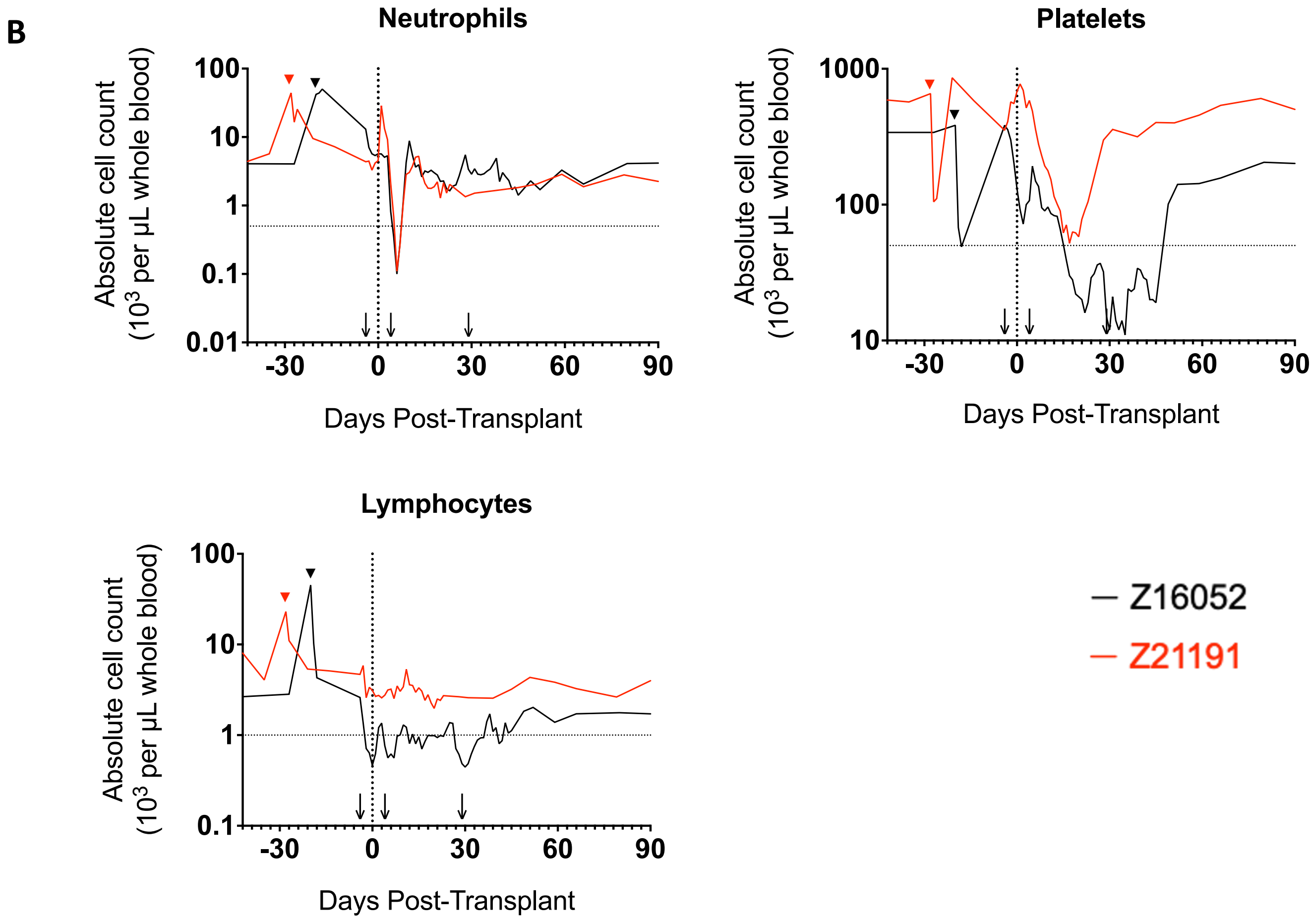

Figure S2. Busulfan pharmacokinetics and post-transplant hematopoietic recovery kinetics in subjects Z16052 and Z21191. (A) Plasma pharmacokinetic clearance profiles of busulfan following intravenous administration. The embedded table summarizes the calculated area under the concentration-time curve (AUC; µM · min), representing total systemic drug exposure over time. (B) Longitudinal peripheral blood cell counts measured before and after autologous HSPC transplantation. Horizontal dashed lines denote clinical toxicity thresholds for neutropenia, thrombocytopenia, and lymphopenia. Black and red triangles indicate the timing of mobilized apheresis harvest (Day −20 for Z16052 and Day −28 for Z21191, respectively). Black arrows along the x-axis denote supportive platelet-rich plasma (PRP) transfusions administered to subject Z16052; transfusions on Days −4 and + 4 were administered for low hematocrit, while transfusion on Day 30 was administered to manage transient post-busulfan thrombocytopenia.

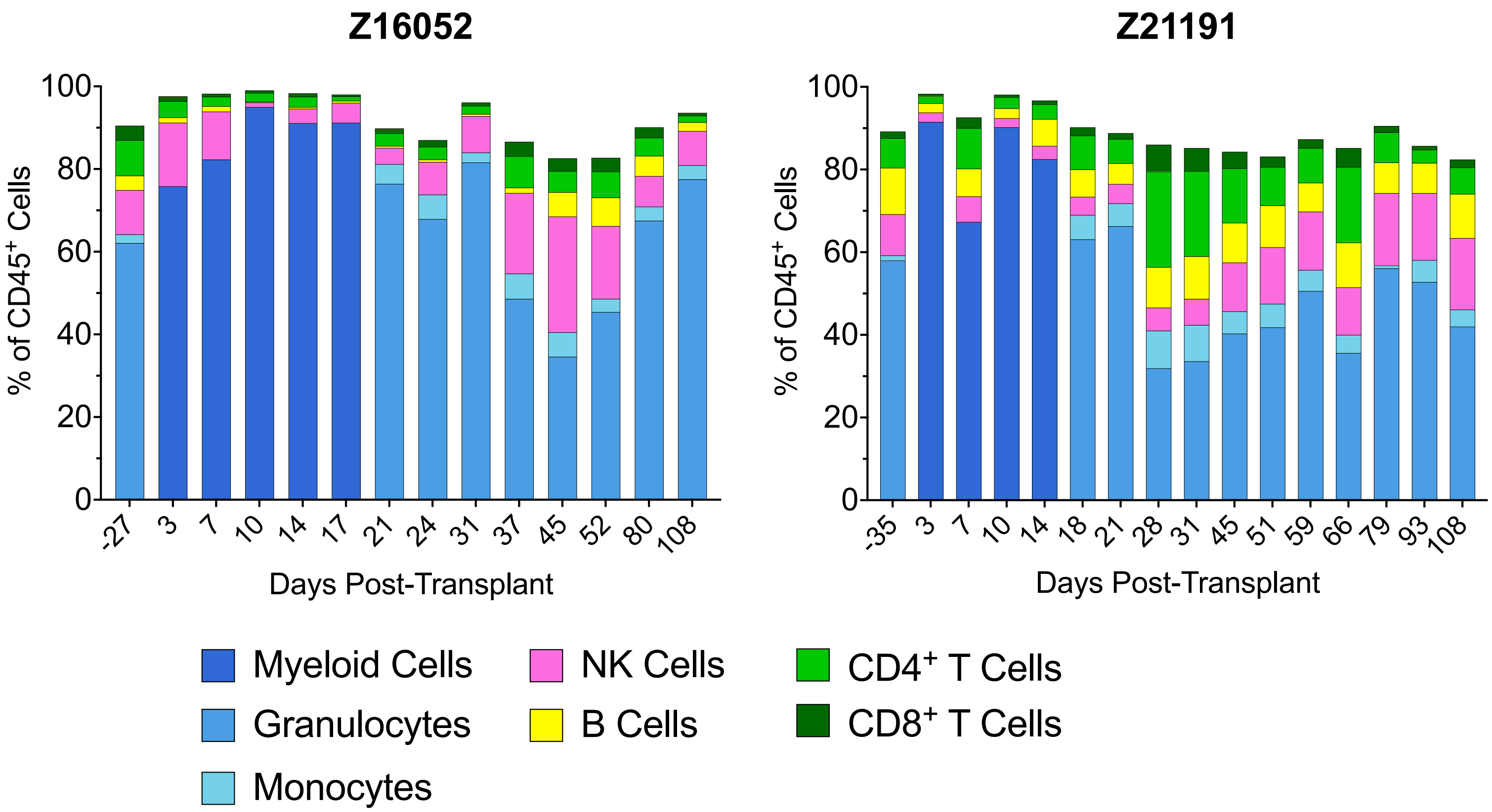

Figure S3. Multi-lineage hematopoietic cell reconstitution. Numbers are presented as percentage of CD45+ cells in peripheral blood after busulfan conditioning and HSC infusion. Myeloid cells: $CD11b^{+}$; Granulocytes: $CD11b^{+}CD14^{-}SSA^{hi}$; Monocytes: $CD11b^{+}CD14^{+}$; NK Cells: $CD11b^{-}CD16^{+}$; B Cells: $CD11b^{-}CD20^{+}$; $CD4^{+}$ T Cells: $CD11b^{-}CD3^{+}CD4^{+}CD8^{-}$; $CD8^{+}$ T Cells: $CD11b^{-}CD3^{+}CD4^{-}CD8^{+}$.

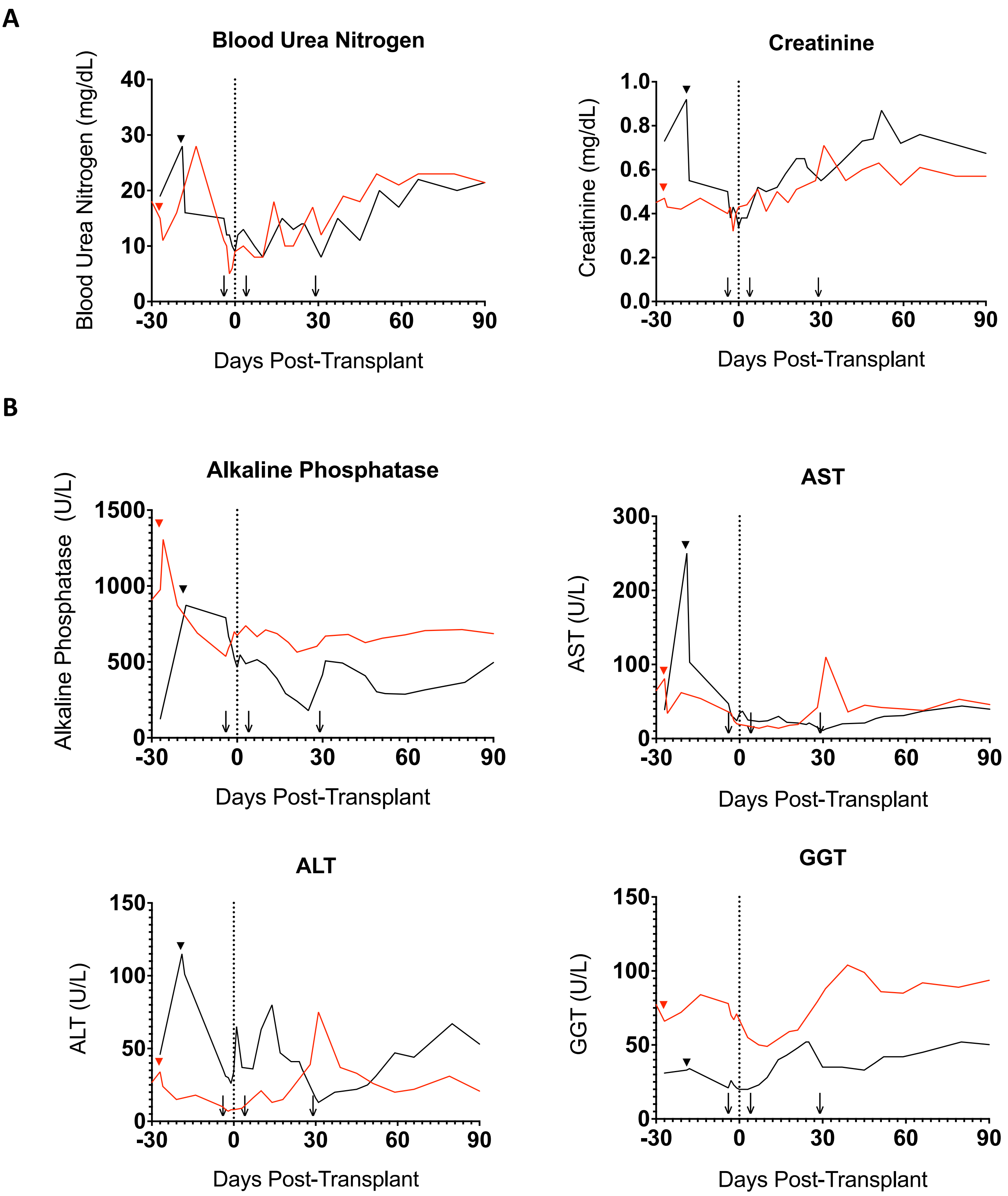

Figure S4. Assessment of renal and hepatic toxicity via longitudinal serum chemistry profiles in busulfan-conditioned pigtail macaques. (A) Serum levels of blood urea nitrogen (BUN) and creatinine in subjects Z16052 and Z21191 before and after autologous HSPC transplantation. Busulfan conditioning was administered during the 4 days preceding transplantation (Days −4 to −1). (B) Serum liver enzyme kinetics, including alkaline phosphatase (ALP), alanine aminotransferase (ALT), aspartate aminotransferase (AST), and gamma-glutamyl transferase (GGT). Black and red triangles denote dates of mobilized apheresis harvest (Day −20 for Z16052 and Day − 28 for Z21191, respectively). Black arrows along the x-axis indicate supportive platelet-rich plasma (PRP) transfusions administered to subject Z16052.

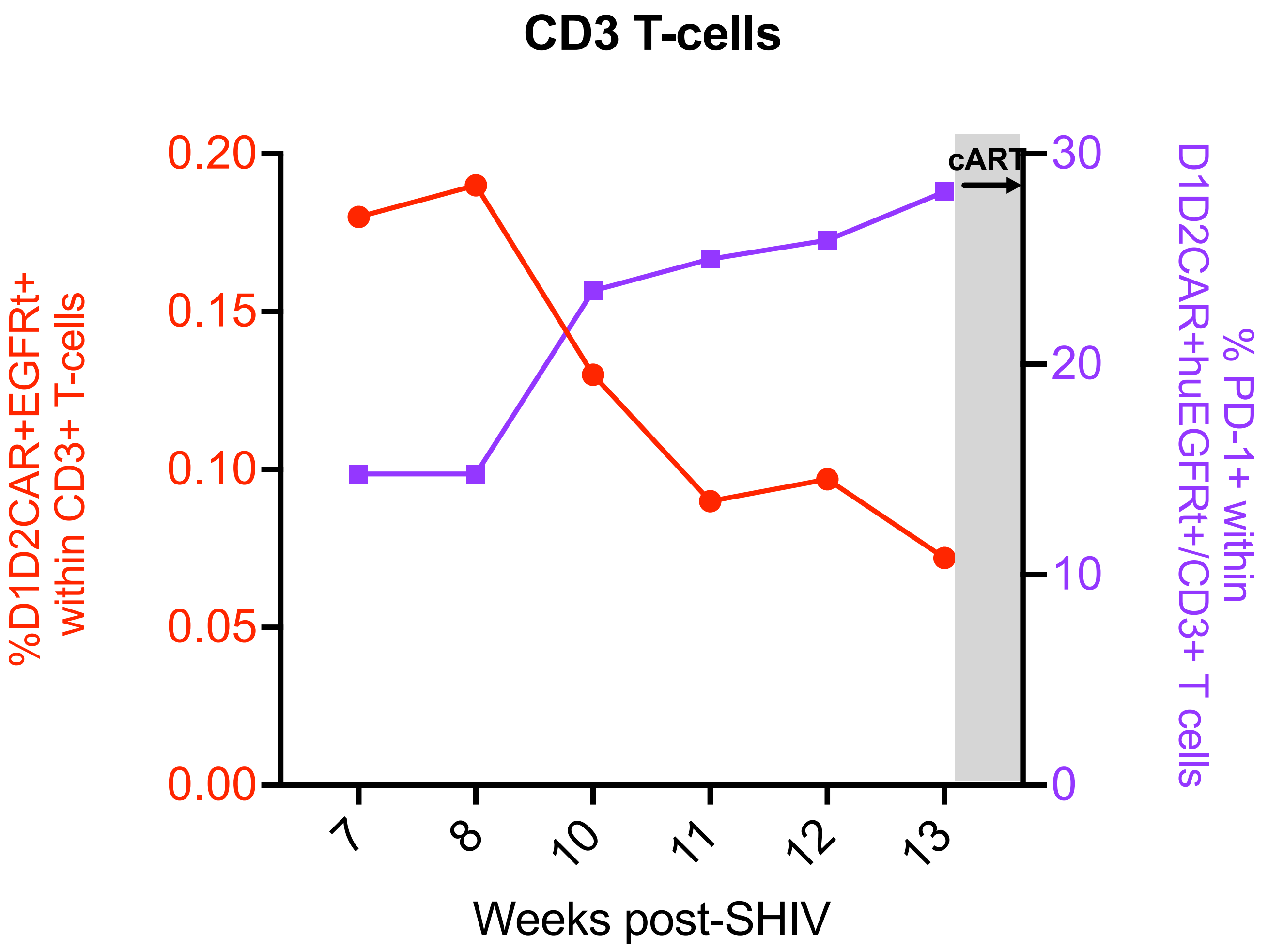

Figure S5. Dynamics of T-cell exhaustion marker PD-1 on D1D2CAR$^+$huEGFRt$^+$ T cells post-SHIV challenge. Kinetics of PD−1 expression relative to D1D2CAR$^+$huEGFRt$^+$ cell frequency in peripheral blood CD3$^+$lymphocytes from animal Z21191 during acute SHIV infection prior to cART administration.

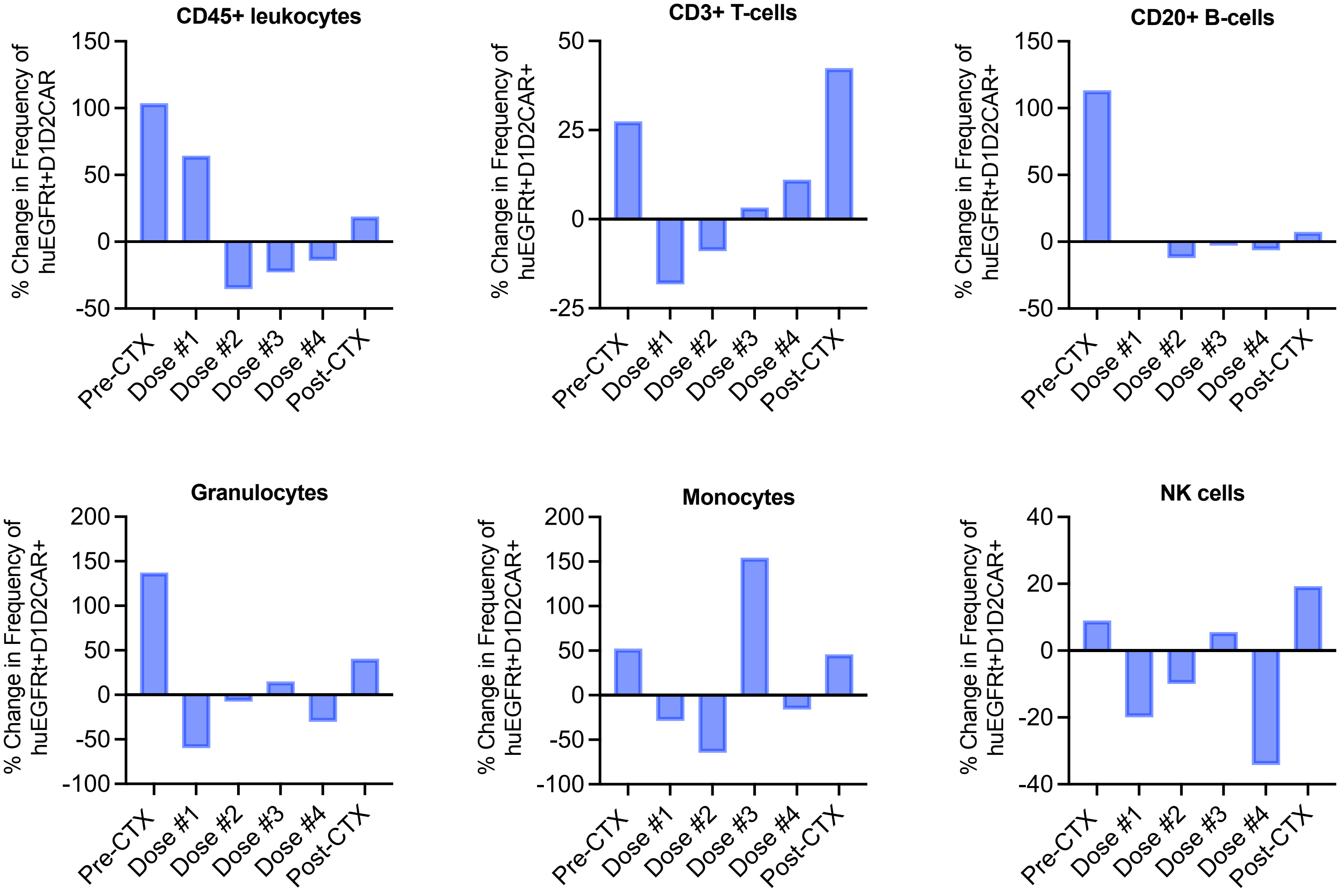

Figure S6: Change in the frequency of huEGFRt$^+$D1D2CAR$^+$ cells across multi-lineage cells. %huEGFRt$^+$D1D2CAR$^+$ double positive cells in the peripheral blood were measured in CD45$^+$ leukocytes, CD3$^+$ T-cells, CD20$^+$ B-cells, CD11b$^+$CD14^{low} granulocytes, CD11b$^+$CD14^{high} monocytes, and NKG2a$^+$ NK cells. Percent change in frequency were calculated as [(%expression post-infusion-%expression pre-infusion)/%expression pre-infusion)x100] between the timepoint preceding (1-3 days before infusion) and 7-10 days following each Cetuximab (CTX) dose and compared with pre-CTX and post-CTX averages.

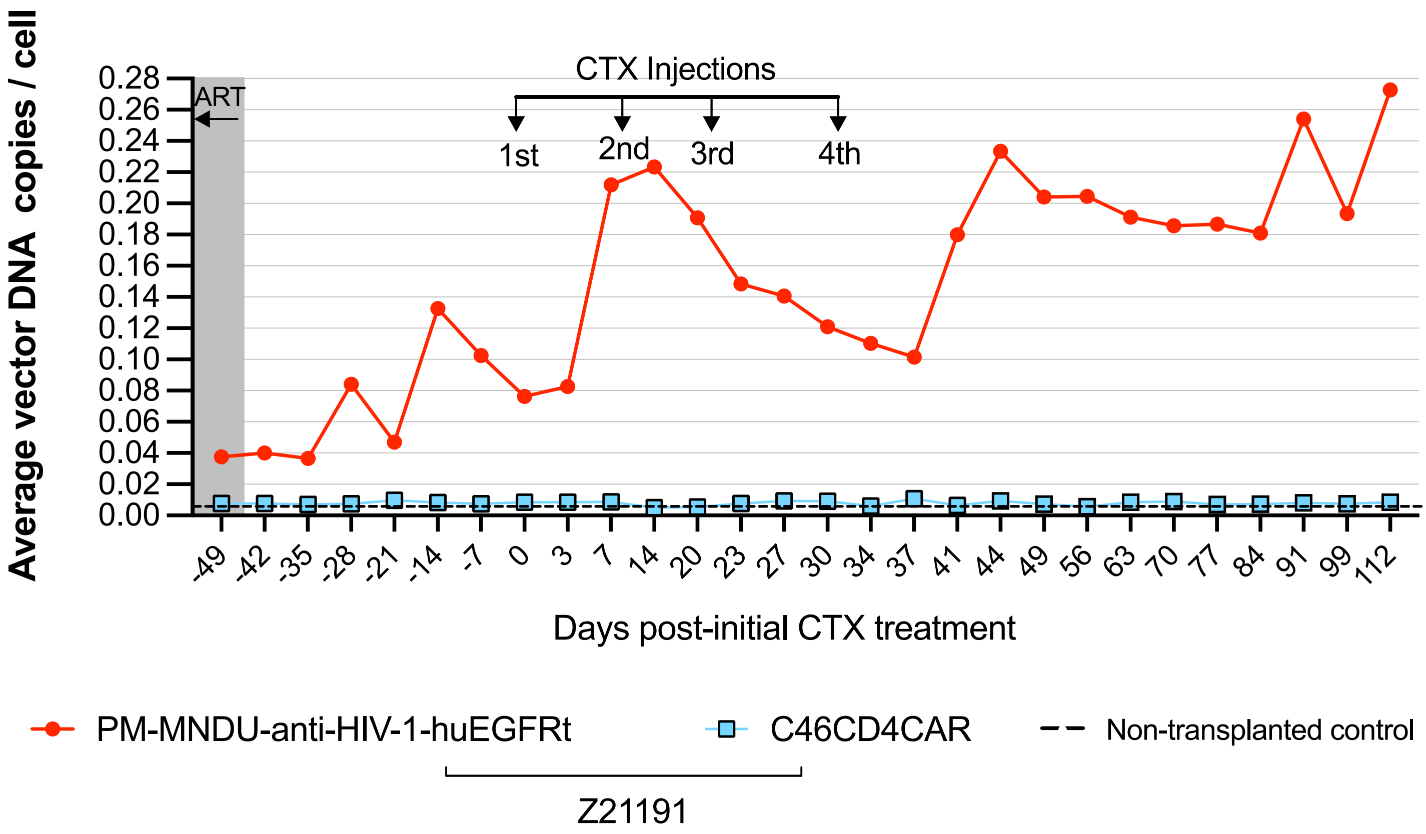

Figure S7. Vector DNA marking dynamics in peripheral blood during Cetuximab (CTX)-mediated negative selection in subject Z21191. Subject Z21191 received a series of four intravenous infusions of the anti-EGFR monoclonal antibody Cetuximab (CTX; three doses at 4 mg/kg followed by a final dose at 5 mg/kg, spaced 1–2 weeks apart). CTX administration induced a transient reduction in PM-MNDU-anti-HIV-1-huEGFRt vector DNA marking following the 3rd and 4th injections. Vector DNA copy numbers per cell were quantified in PBMCs using sequence-specific digital PCR (dPCR) assays for both PM-MNDU-anti-HIV-1-huEGFRt and internal control C46CD4CAR vectors. PBMCs isolated from an untransplanted, uninfected macaque served as a negative control.

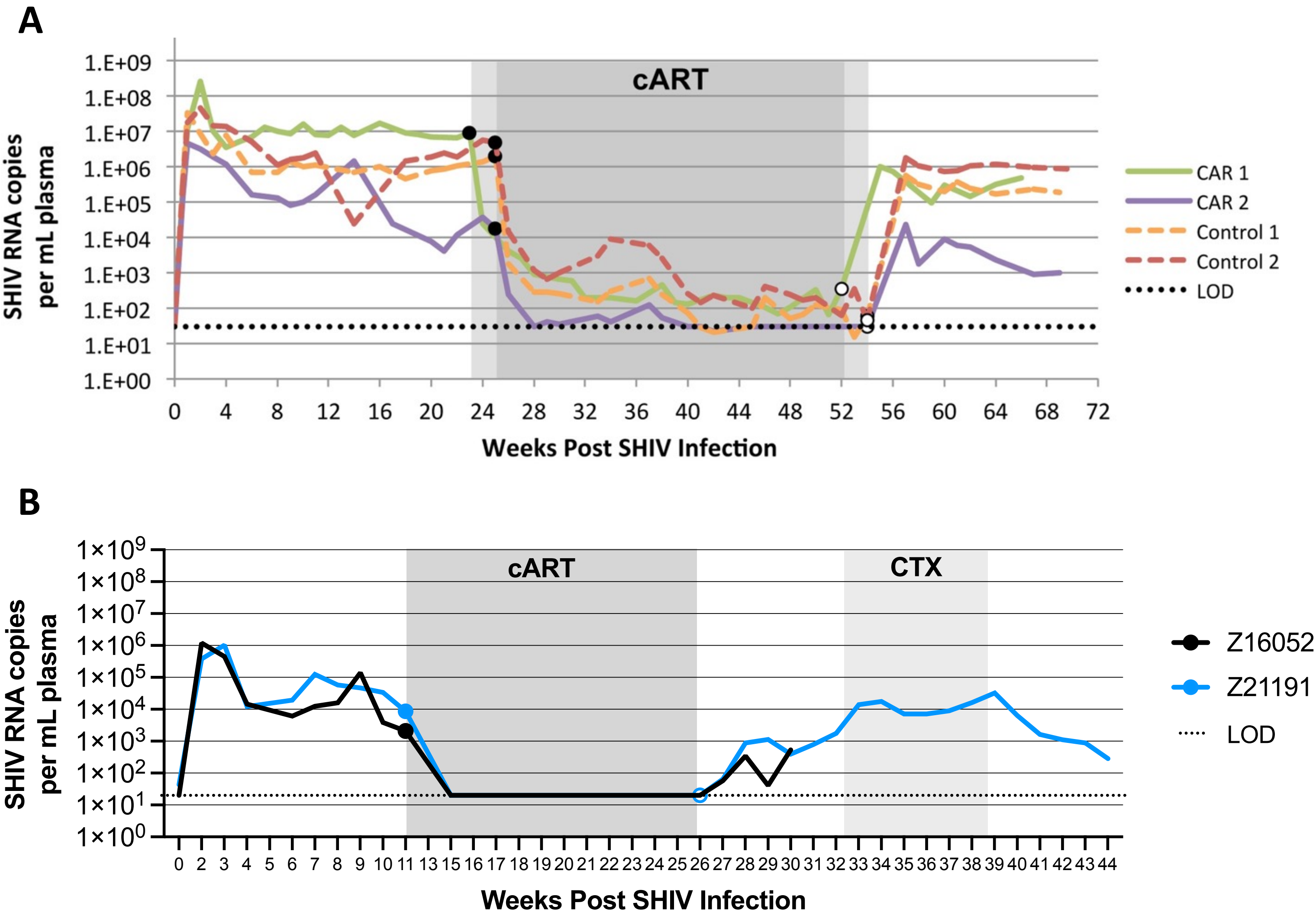

Figure S8. A. Plasma viral loads of C46CD4CAR vector (CAR1 and 2) or a no-HIV-1 gene vector (Control 1 and 2) modified HSPC transplanted pigtail macaques from a previously published study [**27**]. All 4 animals received myeloablative total body irradiation. B. Plasma viral loads of the two pigtail macaques in the current study. Z16052 and Z21191 received myeloablative busulfan. SHIV-1157-ipd3N4 and same anti-retroviral therapy (cART) were used in both studies. LOD: Lower limit of detection of assays.

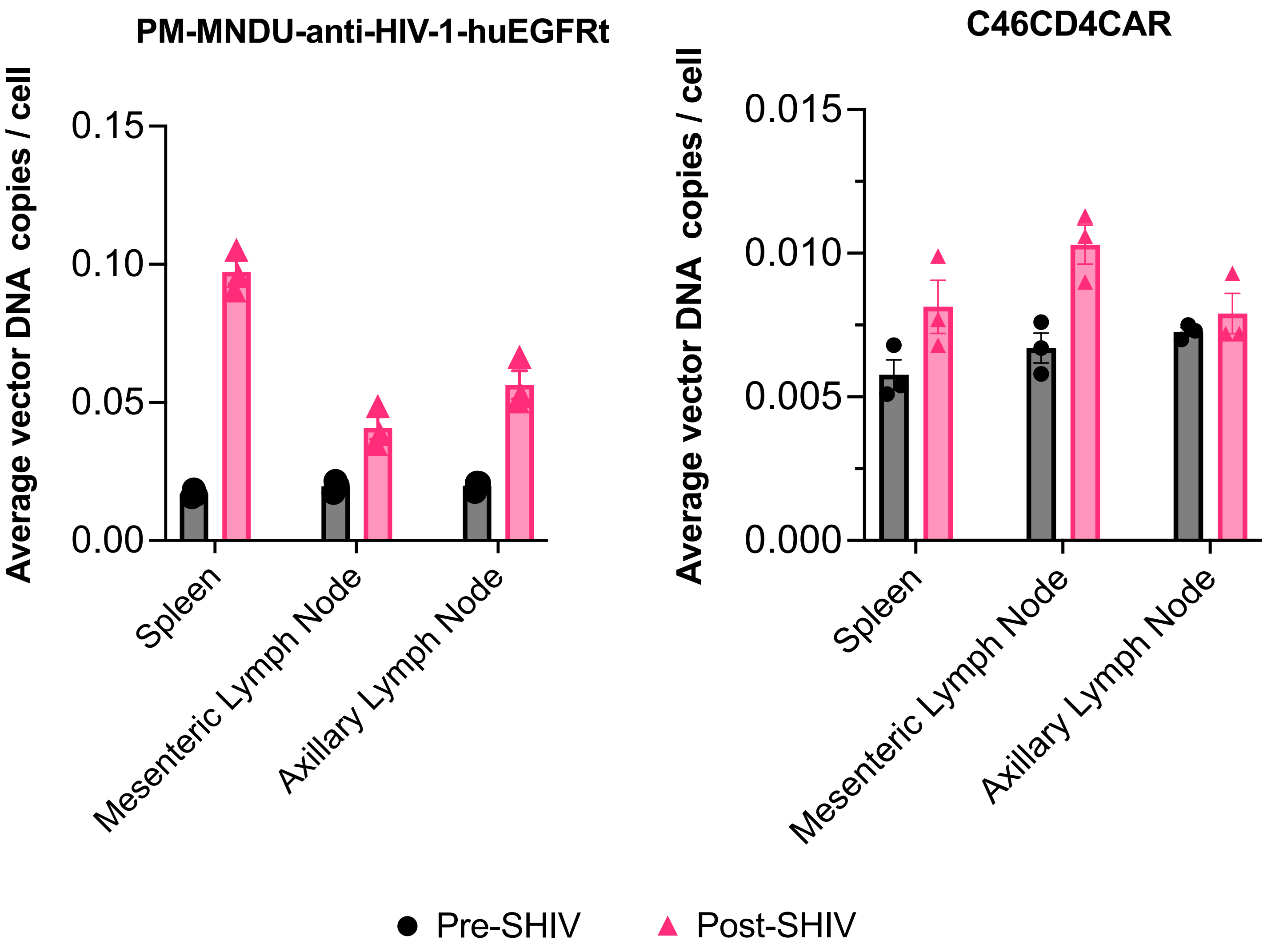

Figure S9. Engraftment and expansion of vector-marked cells across secondary lymphoid tissues in subject Z21191. Longitudinal vector DNA marking levels in single-cell suspensions isolated from the spleen, mesenteric lymph nodes, and axillary lymph nodes. Tissue samples were collected via surgical biopsies pre-SHIV challenge (27 weeks post-HSPC transplant) and at study termination/necropsy post-SHIV infection (86 weeks post-HSPC transplant). Vector DNA copy numbers per cell for PM-MNDU-anti-HIV-1-huEGFRt and internal control C46CD4CAR were quantified using sequence-specific digital PCR (dPCR) assays. Data points represent technical replicates and are expressed as mean ± SEM.

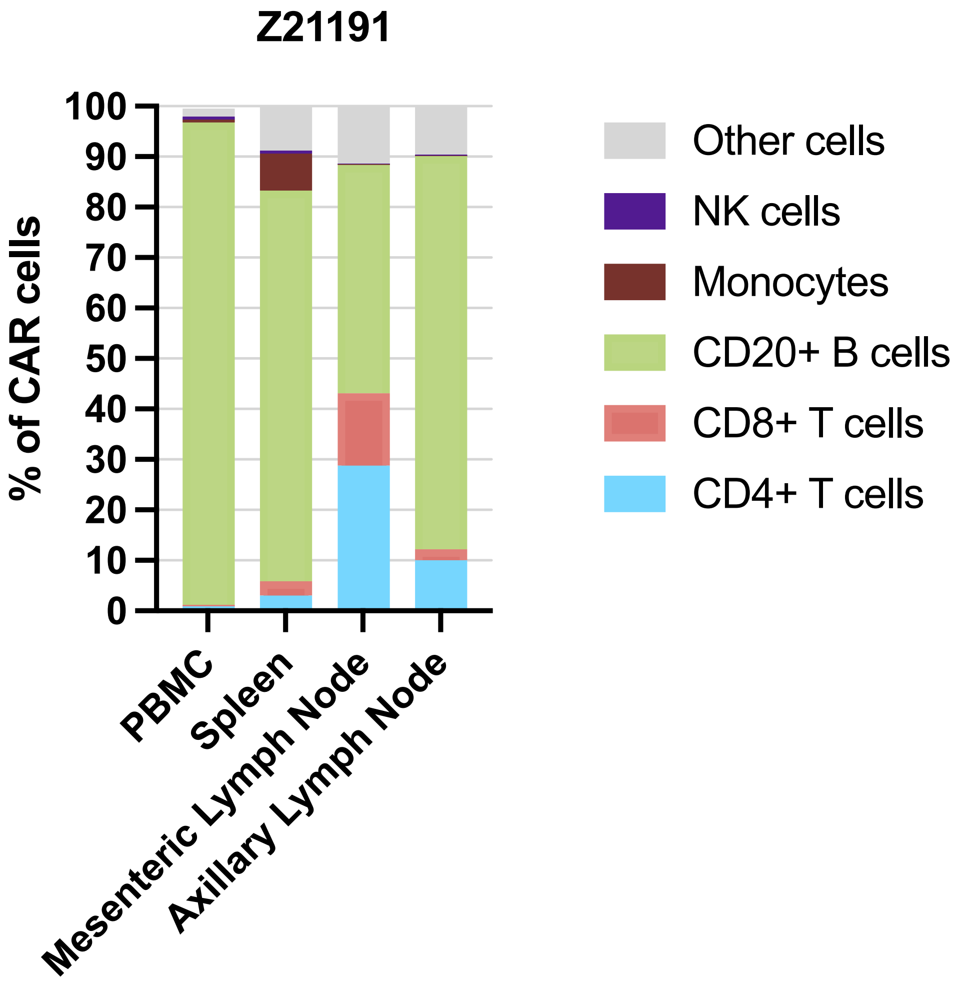

Figure S10. Surface phenotyping of D1D2CAR-expressing cells in PBMC and tissues of Z21191. At necropsy, the indicated PBMC and tissues were collected and measured by flow cytometry. The percentage of D1D2CAR^{+} cells were determined in CD4^{+} or CD8^{+} T cells, CD20^{+} B cells, CD14^{+} monocytes, and NKG2a^{+} NK cells.

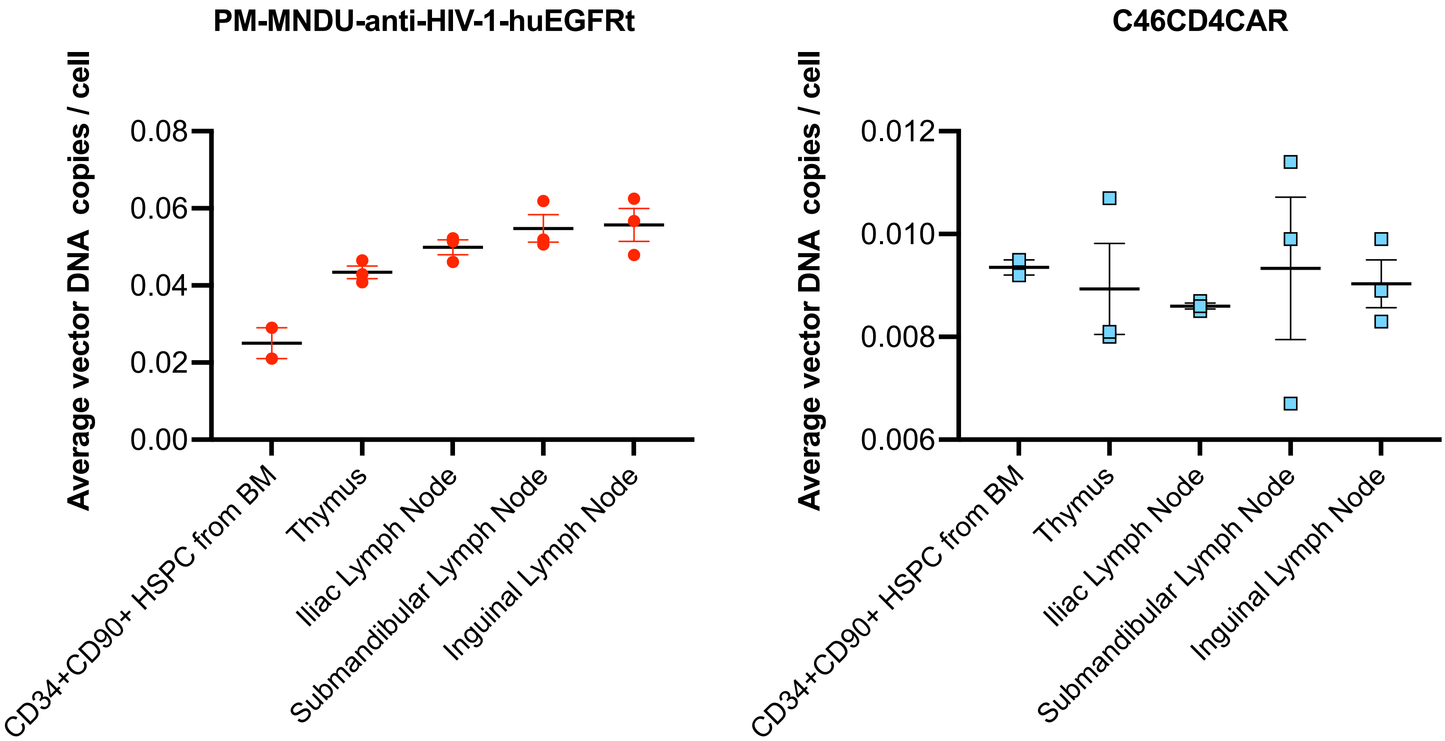

Figure S11. Biodistribution of vector-modified HSPCs and immune cells in bone marrow, thymus, and peripheral lymph nodes of subject Z21191 at necropsy. At study termination (86 weeks post-HSPC transplantation), bone marrow, thymus, and iliac, submandibular, and inguinal lymph nodes were harvested from subject Z21191. Primitive $CD34^{+}CD90^{+}$HSPCs were immunomagnetically enriched from bone marrow cells *ex vivo*. Average vector DNA marking levels for PM-MNDU-anti-HIV-1-huEGFRt and internal control C46CD4CAR were quantified in total tissue cells and enriched HSPCs using sequence-specific digital PCR (dPCR) assays. Data are presented as mean ± SEM.

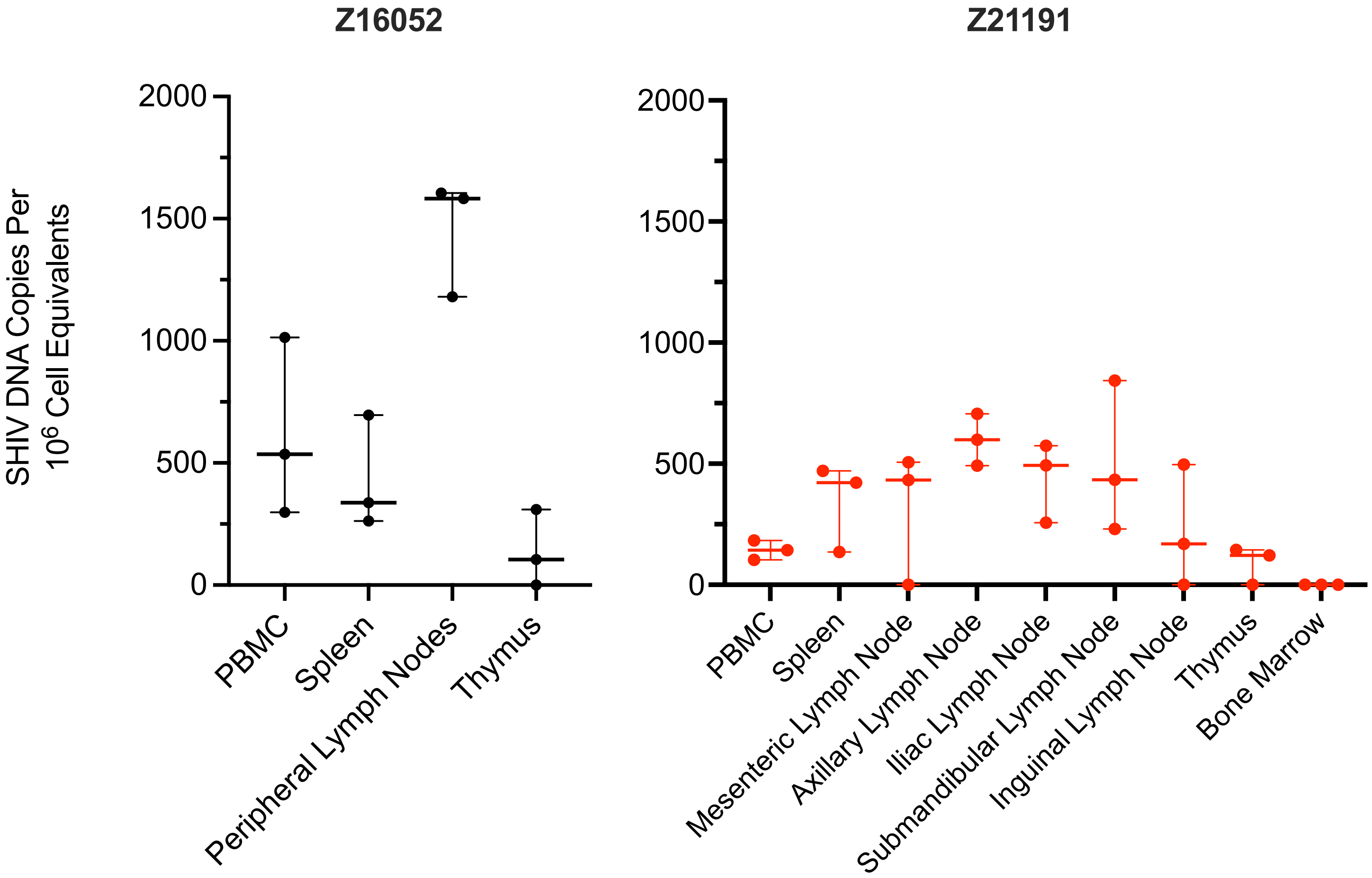

Figure S12. Quantification of SHIV proviral DNA in peripheral blood and tissues. The indicated PBMCs and tissues were collected at necropsy from Z16052 and Z21191. Total genomic DNA were extracted for viral nucleic acid measurements by digital PCR (dPCR). Total SHIV DNA was normalized to a genomic DNA standard (macaque RNaseP p30, MRPP30). Peripheral lymph nodes of Z16052 consist of pooled axillary, iliac, submandibular, and inguinal lymph nodes.
